# Molecular Typing of Gastric Cancer Based on Invasion-Related Genes and Prognosis-Related Features

**DOI:** 10.3389/fonc.2022.848163

**Published:** 2022-06-03

**Authors:** Haonan Guo, Hui Tang, Yang Zhao, Qianwen Zhao, Xianliang Hou, Lei Ren

**Affiliations:** ^1^ Department of Clinical Laboratory, The Affiliated Hospital of Guilin Medical University, Guilin, China; ^2^ Department of Human Resources, The Affiliated Hospital of Guilin Medical University, Guilin, China; ^3^ Central Laboratory, Guangxi Health Commission Key Laboratory of Glucose and Lipid Metabolism Disorders, The Second Affiliated Hospital of Guilin Medical University, Guilin, China

**Keywords:** gastric cancer, invasion, prognosis, TCGA, immune

## Abstract

**Background:**

This study aimed to construct a prognostic stratification system for gastric cancer (GC) using tumour invasion-related genes to more accurately predict the clinical prognosis of GC.

**Methodology:**

Tumour invasion-related genes were downloaded from CancerSEA, and their expression data in the TCGA-STAD dataset were used to cluster samples *via* non-negative matrix factorisation (NMF). Differentially expressed genes (DEGs) between subtypes were identified using the limma package. KEGG pathway and GO functional enrichment analyses were conducted using the WebGestaltR package (v0.4.2). The immune scores of molecular subtypes were evaluated using the R package ESTIMATE, MCPcounter and the ssGSEA function of the GSVA package. Univariate, multivariate and lasso regression analyses of DEGs were performed using the coxph function of the survival package and the glmnet package to construct a RiskScore model. The robustness of the model was validated using internal and external datasets, and a nomogram was constructed based on the model.

**Results:**

Based on 97 tumour invasion-related genes, 353 GC samples from TCGA were categorised into two subtypes, thereby indicating the presence of inter-subtype differences in prognosis. A total of 569 DEGs were identified between the two subtypes; of which, four genes were selected to construct the risk model. This four-gene signature was robust and exhibited stable predictive performance in different platform datasets (GSE26942 and GSE66229), indicating that the established model performed better than other existing models.

**Conclusion:**

A prognostic stratification system based on a four-gene signature was developed with a desirable area under the curve in the training and independent validation sets. Therefore, the use of this system as a molecular diagnostic test is recommended to assess the prognostic risk of patients with GC.

## Background

Gastric cancer (GC) is considered the most common malignancy of the digestive system and the third leading cause of cancer-related deaths worldwide (1). It is considered a public health concern worldwide, especially in developing countries, owing to its high incidence and mortality rates (2). The National Cancer Center of China show that GC ranked second in terms of the incidence of malignant tumours in 2015, with approximately 403,000 cases and 291,000 deaths (3). Although the development of integrated treatment modalities, including surgery, radiotherapy and immunotherapy, has improved the survival of patients with GC in recent years, the 5-year survival rate remains <30% (4). Moreover, these patients are susceptible to multiple forms and different degrees of invasion and metastasis after treatment, primarily blood, lymphatic and peritoneal dissemination metastases (5). Therefore, exploratory studies aimed at optimising the prognostic predictors of GC are warranted.

Invasion and metastasis constitute two important features of malignancy and are the leading causes of cancer-related deaths. Despite the genetic heterogeneity of GC, several biological factors affecting GC invasion have been identified in recent years, such as ADAMTS5 (6), HOXD9 (7), MTMR2 (8) and SIRT2 (9), which can be used as indicators of patient prognosis. However, because individual clinical biomarkers may be influenced by multiple factors, predictive accuracy can be improved by establishing a combination signature comprising the most ideal candidate biomarkers.

Reanalysis of global cancer data resources, aided by the development of high-throughput sequencing and public databases, has reduced economic expenditure and any bias introduced by sample, geographical and ethnic factors. The seven-gene signature constructed by Xu et al. (10) based on immune-related genes can be used to predict the overall survival of men with GC. The five-gene signature constructed by Zhao et al. (11) based on genes related to the cell cycle can be used to predict the prognosis of patients with GC. The prognostic model constructed by Peng et al. (12) based on DNA methylation-related genes plays an important role in the stratification of patients with GC. However, to the best of our knowledge, no study has comprehensively analysed the vital functions of invasion-related genes in GC.

In this study, the gene expression profile data from public databases, such as TCGA and GEO, were used to construct molecular subtypes of GC based on tumour invasion-related genes. In addition, these data were used to assess the correlation among the molecular subtypes, prognosis and clinical features of GC. Furthermore, a prognostic risk model was constructed using differentially expressed genes (DEGs) among the STAD molecular subtypes. This model performed better in terms of predicting the prognosis of STAD samples. The results were further validated to ensure desirable performance using the GEO gene expression dataset.

## Methodology

### Data Source and Pre-Processing

The RNA-Seq data of patients with GC and their clinical follow-up information were downloaded from TCGA database, whereas the expression data and clinical information of the GEO microarray datasets GSE66229 and GSE26942 with time-to-live (TTL) information were downloaded from the GEO database. The invasion-related gene set was obtained from CancerSEA (13), which included a total of 97 genes (**Table S1**).

The RNA-Seq data of the TCGA-STAD cohort were processed as follows: 1) samples without clinical follow-up information were excluded; 2) samples without TTL data were excluded; 3) samples without information related to patient survival status were excluded; 4) the Ensembl IDs were converted to Gene Symbol and 5) the median value was recorded if there were multiple Gene Symbol expressions. The GEO dataset was processed as follows: 1) samples without clinical follow-up information were excluded; 2) samples without information regarding the TTL and survival status of patients were excluded; 3) the probe IDs were converted to Gene Symbol; 4) probe IDs corresponding to multiple genes were excluded and 5) the median value was recorded if there were multiple Gene Symbol expressions. After preprocessing the data, a total of 353, 202 and 300 samples were selected from the TCGA-STAD, GSE26942 and GSE66229 datasets, respectively. The clinical information of these samples is presented in **Table 1**.

**Table 1:** Sample information.

Clinical Features	TCGA-STAD	GSE26942	GSE66229
OS			
Alive	210	114	148
Dead	143	88	152
**T Stage**			
T1	18		
T2	74		
T3	163		
T4	94		
TX	4		
**N Stage**			
N0	103		
N1	96		
N2	72		
N3	71		
NX	11		
**M Stage**			
M0	314		
M1	23		
MX	16		
**Stage**			
I	48		
II	109		
III	146		
IV	35		
X	15		
**Grade**			
G1	9		
G2	128		
G3	207		
GX	9		
**Gender**			
Male	228		
Female	125		
**Age**			
≤65	158		
>65	192		
Unknown	3		

### Non-Negative Matrix Factorization (NMF) Algorithm

The expression data of 97 invasion-related genes were extracted from TCGA database, and the STAD samples were clustered using NMF. The ‘brunet’ criterion was selected for the method along with 100 iterations. The number of clusters *k* was set from 2 to 10. The average contour width of the common membership matrix was determined *via* the R package ‘NMF’. The minimum membership of each subclass was set to 10. The stability of clusters obtained *via* NMF was reflected using the value of the cophenetic correlation, which was between 0 and 1. The larger the value, the greater the cluster stability. Furthermore, smaller values of residual sum of squares (RSS)—used to reflect the clustering performance of the model—were indicative of the better clustering performance of the model. Optimal cluster numbers were determined based on the cophenetic, dispersion and silhouette metrics. Through the above algorithm, the samples are divided into different molecular subtypes.

### Identification and Functional Analysis of DEGs

DEGs between molecular subtypes were identified, and volcano plots demonstrating these DEGs were plotted using the limma package (14), with the thresholds set as FDR < 0.05 and |log2FC| > 1. KEGG pathway and GO functional enrichment analyses of the DEGs were performed using the R package WebGestaltR (v0.4.2).

### Immune Scores Between Molecular Subtypes

The three scores, namely, the immune score, stromal score and estimate score were assessed using the R package ESTIMATE, whereas 10 immune cell scores were assessed using MCPcounter, and 28 immune cell scores were assessed using the ssGSEA function of the GSVA package (15). Molecular subtypes were compared based on differences in their immune scores.

### Construction of a Risk Model

The 353 samples in TCGA dataset were divided into the training and validation sets. Random assignment bias, which influences the stability of subsequent modelling, was avoided by randomly grouping all samples 100 times with replacements in advance. In addition, group sampling was performed at a ratio of 1:1 (training set:validation set), with 176 samples in the training set and 177 samples in the validation set. Univariate Cox proportional risk regression was performed on DEGs and survival data of molecular subtypes in the training set using the coxph function of the R package survival; a p-value of <0.05 was considered the threshold for screening prognosis-related genes. Lasso regression analysis of the identified genes was performed using the R package glmnet to reduce the number of genes in the risk model (16). Eventually, a model was constructed using the 5-fold cross-validation method.

### GSEA

The relationship between the RiskScore of different samples and biological functions was examined *via* single-sample GSEA using the R package GSVA. The ssGSEA scores of each function, which corresponded to each sample, were obtained by calculating the scores of each sample on different functions. After performing additional calculations related to the correlation between these functions and RiskScores, functions with a correlation coefficient of >0.45 were selected.

## Results

### Molecular Subtypes of STAD Based on Invasion-Related Genes

**Figure 1:**
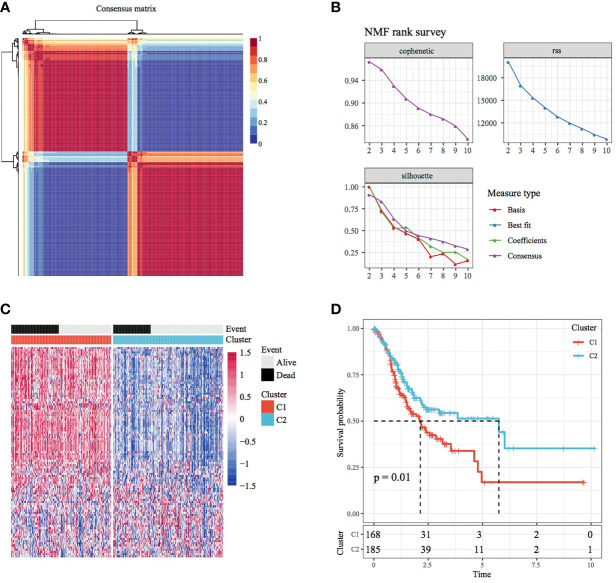
Molecular subtypes of STAD based on invasion-related genes. **(A)** Consensus map of NMF clustering. **(B)** Distribution of cophenetic, RSS and dispersion with a rank of 2–10. **(C)** Heat map of 40 prognosis-related gene clusters. **(D)** Prognostic survival curve of STAD in molecular subtypes.

The NMF algorithm was used for clustering TCGA-STAD samples, with the optimal number of clusters selected as 2 (**Figures 1A, B**). The expression of prognosis- and invasion-related genes in the two subtypes (Cluster 1 and Cluster 2) is shown in **Figure 1C**, which demonstrates that the expression of invasion-related genes was different in the Cluster 1(C1) and Cluster 2(C2) subtypes. In addition, most genes were highly expressed in the C1 subtype. On analysing the relationship between the two subtypes and prognosis, a difference was found in TTL between the C1 and C2 subtypes (**Figure 1D**, log-rank *p* < 0.05).

### Identification and Functional Analysis of DEGs Between Molecular Subtypes

**Figure 2:**
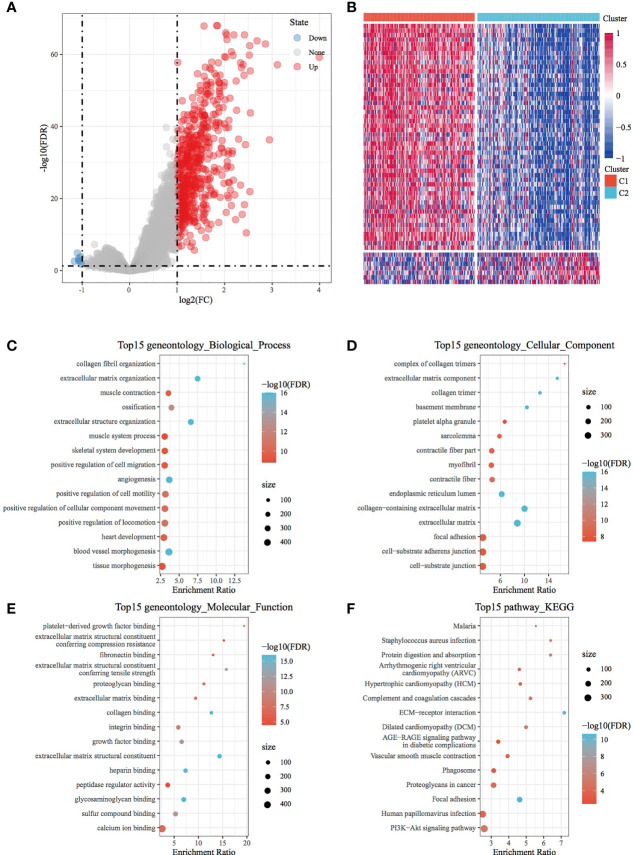
Identification and functional analysis of DEGs between molecular subtypes. **(A)** Volcano map of DEGs between the C1 and C2 subtypes. **(B)** Heat map of DEGs between the C1 and C2 subtypes. **(C)** BP annotation map of differentially upregulated genes in the molecular subtypes. **(D)** CC annotation map of differentially upregulated genes in the molecular subtypes. **(E)** MF annotation map of differentially upregulated genes in the molecular subtypes. **(F)** KEGG annotation map of differentially upregulated genes in the molecular subtypes.

A total of 569 DEGs were observed between the C1 and C2 subtypes after filtering data according to a threshold (**Table S2**). Of these DEGs, 562 were upregulated and 7 were downregulated. This finding consequently highlighted the dominance of upregulated differential expression between the C1 and C2 subtypes (**Figure 2A**). A heat map demonstrating the 50 most upregulated DEGs and all downregulated DEGs is presented in **Figure 2B**. Furthermore, GO functional enrichment analysis of DEGs revealed that 456, 54 and 49 GO functional pathways were annotated to BP, CC and MF, respectively, with differences (FDR < 0.05) (**Figures 2C, D**; the first 15 annotations are shown in **Figure 2E**). In addition, KEGG pathway enrichment analysis revealed the presence of 15 annotations (FDR < 0.05) (**Figure 2F**), which also included ECM–receptor interaction, focal adhesion, PI3K–Akt signalling pathway, proteoglycans in cancer and other tumour-related pathways.

### Comparison of Immune Scores, Clinical Features and Immune Subtypes Between Molecular Subtypes

**Figure 3:**
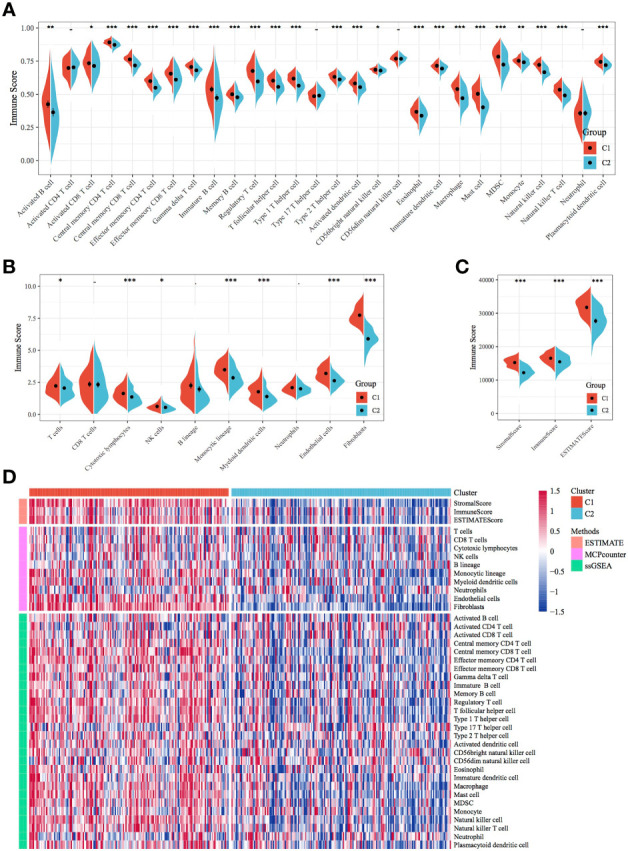
Comparison of immune scores and immune subtypes between molecular subtypes. **(A)** Comparison of immune scores calculated using ssGSEA between the molecular subtypes of TCGA dataset. **(B)** Comparison of immune scores calculated using MCPcounter between the molecular subtypes of TCGA dataset. **(C)** Comparison of immune scores calculated using ESTIMATE between the molecular subtypes of TCGA dataset. **(D)** Clustering heatmap of molecular subtypes of immune infiltration patterns between different algorithms.*p < 0.05, **p < 0.01, ***p < 0.001.

**Figure 4:**
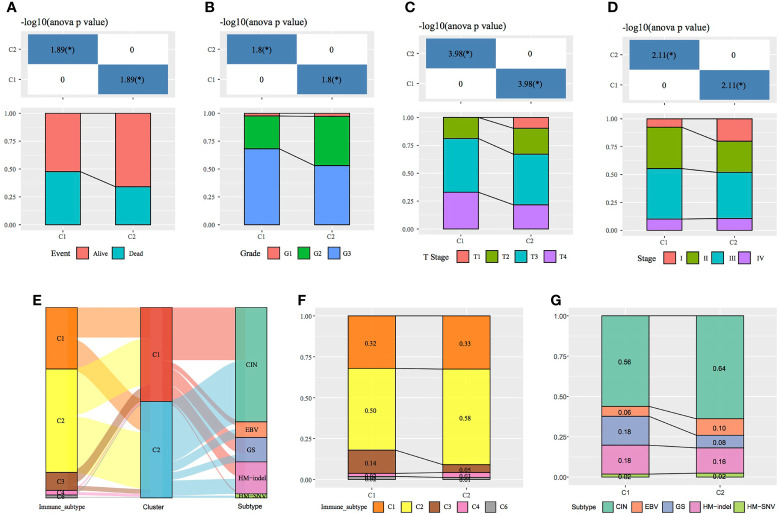
Comparison of clinical features between molecular subtypes. **(A–D)** Comparison of the distribution of different clinical features between the two molecular subtypes in TCGA dataset. **(E)** Sankey diagram demonstrating the comparison between the molecular subtypes and existing subtypes. **(F)** Comparison between the molecular subtypes established in this study and immune subtypes in existing TCGA cancers. **(G)** Comparison between the molecular subtypes established in this study and the four molecular subtypes in TCGA gastric cancer data.*p < 0.05, **p < 0.01, ***p < 0.001.

Immune scores were calculated and compared between molecular subtypes using the ESTIMATE and MCPcounter R software packages and the ssGSEA function of the GSVA package. The results showed that the immune scores of the C1 subtype were higher than those of the C2 subtype (**Figures 3A–C**). A heat map demonstrating the immune scores of the two subtypes is shown in **Figure 3D**. Furthermore, the distribution of different clinical features in the two molecular subtypes was analysed, which revealed differences in the surviving fraction of the two subtypes. The C1 subtype had a higher proportion of death and a poor prognosis (**Figure 4A**). Inter-subtype grading proportions were notably different, with a higher proportion of the more differentiated G3 observed in the poorly prognostic C1 subtype (**Figure 4B**). T staging proportion was different between the two subtypes, with the poorly prognostic C1 subtype having higher proportions of T2, T3 and T4 samples (**Figure 4C**). Staging proportions were different between the two subtypes, with a higher proportion of stage II, III and IV samples observed in the poorly prognostic C1 subtype (**Figure 4D**).

A total of 33 cancers have been previously examined in TCGA project, and the tumours have been categorised into six immune subtypes as follows: C1 (wound healing), C2 (IFN-γ dominant), C3 (inflammation), C4 (lymphocyte depletion), C5 (immunologically silent) and C6 (TGF-beta dominance). The C1, C2 and C6 subtypes correlate with a poor prognosis (17). The following four molecular subtypes of GC have been reportedly identified through molecular evaluation of 295 primary GC samples from TCGA database: chromosomal instability (CIN), Epstein–Barr virus (EBV) positivity, genetic stability (GS) and microsatellite instability (MSI) (18). In this study, further comparison of the sample distribution between these molecular subtypes and the two subtypes analysed in this study (**Figures 4E–G**) demonstrated that the C2 subtype had the highest proportion of immune subtype C2 (IFN-γ dominant), (58%). However, the proportion of immune subtype C3 (inflammation) (14%) was higher in the C1 subtype than in the C2 subtype. Compared with the previously established molecular subtypes of GC, the C2 subtype in the present study comprised the highest proportion of the CIN subtype, whereas the proportion of the GS subtype was higher in the C1 than in the C2 subtype.

### Construction and Evaluation of a Four-Gene Signature

**Figure 5:**
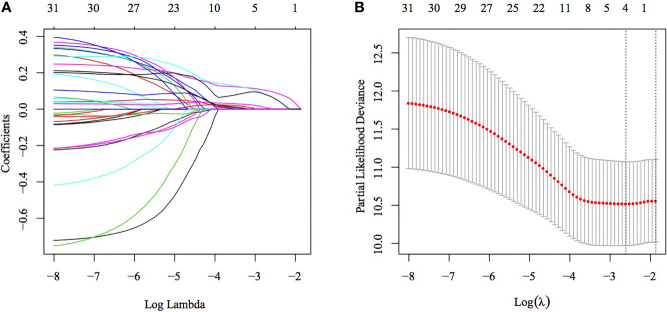
Construction of a multigene model using lasso–Cox regression. **(A)** Trajectory of each independent variable, wherein the horizontal axis represents the log value of the independent variable lambda and the vertical axis represents the coefficient of the independent variable. **(B)** Confidence interval under each lambda.

Univariate Cox regression analysis of TCGA training set for screening DEGs between the C1 and C2 subtypes revealed that 32 genes correlated with prognosis (**Table S3**). The number of genes was further reduced using lasso–Cox regression analysis (**Figure 5A**), in which a gradual increase in lambda resulted in a gradual increase in the number of corresponding independent variable coefficients tending to zero. A model was constructed using 5-fold cross-validation. Confidence intervals (CIs) for each lambda (**Figure 5B**) showed that the model was optimal when the value of lambda was 0.07371266. Therefore, four genes (SERPINE1, MATN3, AMIGO2 and NOX4) with a lambda of 0.0737 were selected as target genes for the subsequent process. The formula of the final four-gene signature is as follows: RiskScore = 0.146 * SERPINE1 + 0.171 * MATN3 + 0.06 * AMIGO2 + 0.149 * NOX4.

On comparing the expression of these four genes in TCGA dataset, it was found that the expression of these genes was higher in tumour samples than in healthy tissues. Moreover, the expression of SERPINE1 and NOX4 was significantly different between the two sample types (**Supplementary Figure 1**). Subsequently, we compared the expression of these genes in different TNM stages (**Supplementary Figure 2**).

**Figure 6:**
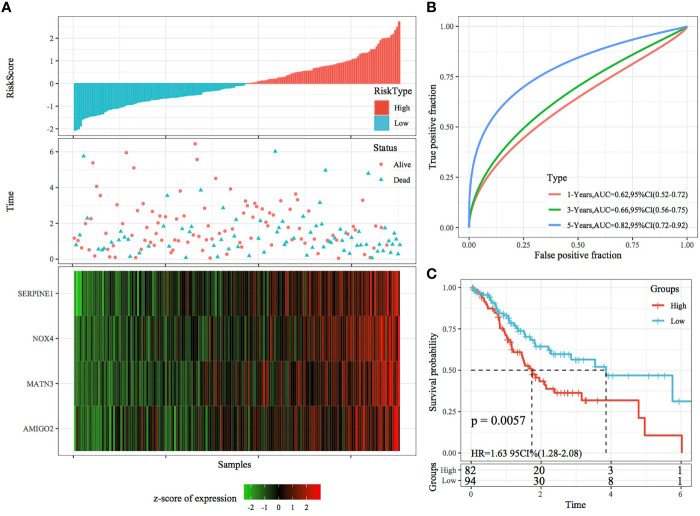
Validation of the four-gene signature in the training set. **(A)** RiskScore, survival status and the expression of four genes in TCGA training set. **(B)** ROC curve and AUC of the four-gene signature. **(C)** KM curve demonstrating survival predicted by the four-gene signature in the training set.

The RiskScore of each sample was calculated according to gene expression, and the RiskScore distribution of samples was plotted (**Figure 6A**). The TTL of STAD samples with high RiskScores was significantly shorter than that of samples with low RiskScores, suggesting that a high RiskScore was associated with a relatively poor prognosis. In terms of changes in the expression of the four genes with an increasing RiskScore, high expression of SERPINE1, MATN3, AMIGO2 and NOX4 was correlated with a high risk of STAD. Furthermore, ROC analysis was performed using the R package timeROC (**Figure 6B**) to evaluate the prognostic efficiency of the RiskScore at 1, 3 and 5 years. The results revealed that the RiskScore model had a high area under the ROC curve (AUC). Evaluation of the z-score revealed that samples with a RiskScore of >0 were classified as high risk, whereas those with a RiskScore of <0 were classified as low risk. Among these samples, 82 were classified as high risk, whereas 94 were classified as low risk, and KM curves demonstrated differences between the high- and low-risk groups (p < 0.01; **Figure 6C**).

### Robustness of the Risk Model Validated Using Internal and External Datasets

**Figure 7:**
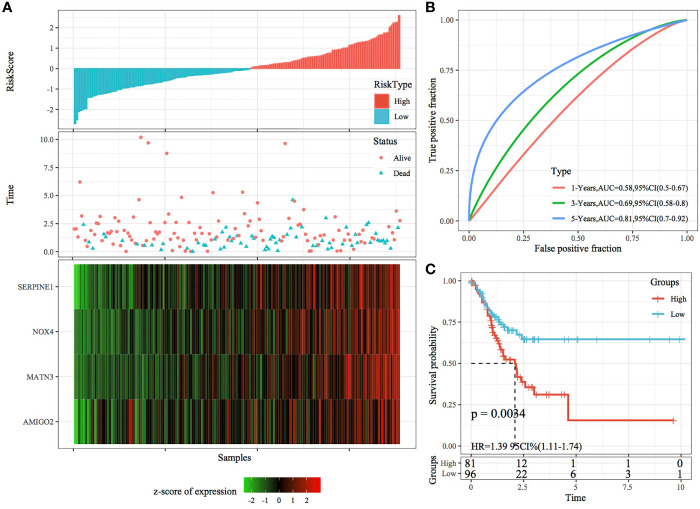
Validation of the four-gene signature in the validation set. **(A)** RiskScore, survival status and the expression of four genes in TCGA validation set. **(B)** ROC curve and AUC of the four-gene signature. **(C)** KM curve demonstrating survival predicted by the four-gene signature in the validation set.

**Figure 8:**
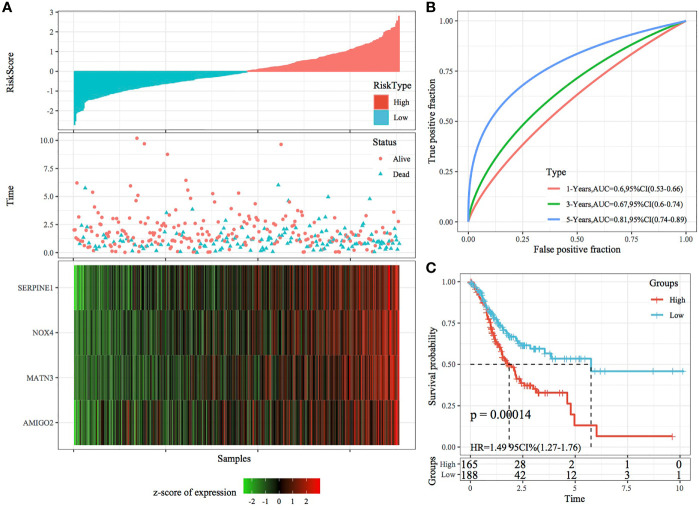
Validation of the four-gene signature in the whole TCGA dataset. **(A)** RiskScore, survival status and the expression of four genes in the whole TCGA dataset. **(B)** ROC curve and AUC of the four-gene signature. **(C)** KM curve demonstrating survival predicted by the four-gene signature in the whole TCGA dataset.

The robustness of the RiskScore model was validated using the same coefficients as the training set, and the RiskScore of each sample was calculated based on gene expression. **Figures 7A**, **8A** demonstrate the RiskScore distribution of TCGA validation set and the whole dataset. According to these figures, the TTL of STAD samples with high RiskScores was shorter than that of samples with low RiskScores, indicating that samples with high RiskScores had a poorer prognosis. As mentioned earlier, high expression of SERPINE1, MATN3, AMIGO2 and NOX4 indicated a high risk for STAD, which was consistent with results obtained in the training set. ROC analysis was performed using the R package timeROC to analyse the prognostic efficiency of the RiskScores at 1, 3 and 5 years (**Figures 7B**, **8B**). In TCGA validation set, 81 and 96 samples were classified as high and low risk, respectively. KM curves demonstrated differences between the high- and low-risk groups (p < 0.01; **Figure 7C**). In the whole dataset, 165 samples were classified as high risk, whereas 188 samples were classified as low risk, and KM curves demonstrated differences between the high- and low-risk groups (p < 0.001; **Figure 8C**).

**Figure 9:**
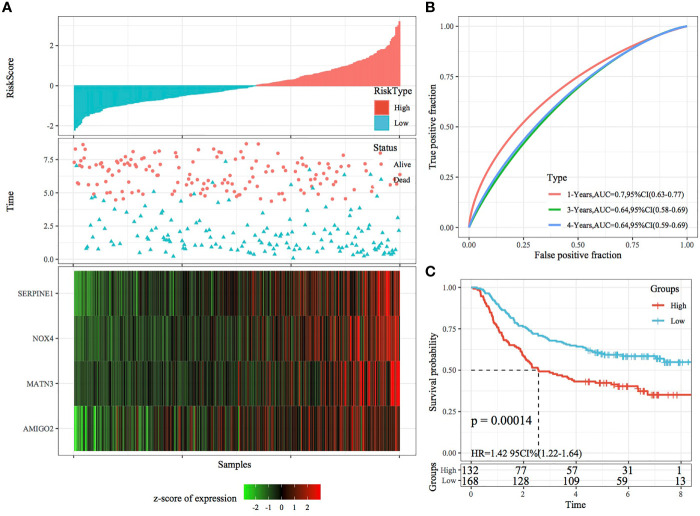
Validation of the four-gene signature in the GSE66229 dataset. **(A)** RiskScore, survival status and the expression of four genes in the independent validation dataset GSE66229. **(B)** ROC curves and AUC of the four-gene signature. **(C)** KM curve demonstrating survival predicted by the four-gene signature in the independent validation dataset GSE66229.

**Figure 10:**
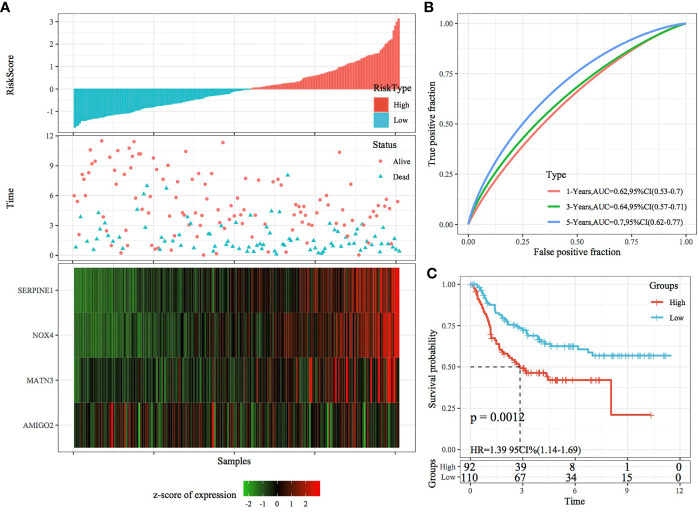
Validation of the four-gene signature in the GSE26942 dataset. **(A)** RiskScore, survival status and the expression of four genes in the independent validation dataset GSE26942. **(B)** ROC curve and AUC of the four-gene signature. **(C)** KM curve demonstrating survival predicted by the four-gene signature in the independent validation dataset GSE26942.

In the external datasets GSE66229 and GSE26942, we used models and coefficients similar to those used in the training set to calculate the RiskScore of each sample according to gene expression. **Figures 9A**, **10A** demonstrate the RiskScore distribution of the independent validation datasets GSE66229 and GSE26942, respectively. As shown in the two figures, the TTL of STAD samples with high RiskScores was shorter than that of samples with low RiskScores, indicating that samples with high RiskScores had a relatively poor prognosis. This result was consistent with that observed in the TCGA training set. **Figures 9B**, **10B** demonstrate the prognostic efficiency of the RiskScore in the two datasets at 1, 3 and 5 years. In the GSE66229 dataset, 132 and 168 samples were classified as high and low risk, respectively, and KM curves demonstrated significant differences between the high- and low-risk groups (p < 0.001; **Figure 9C**). In the GSE26942 dataset, 92 and 110 samples were classified as high and low risk, respectively, and KM curves demonstrated significant differences between the high- and low-risk groups (p < 0.01; **Figure 10C**).

### RiskScore and Prognostic Analysis of Clinical Features

**Figure 11:**
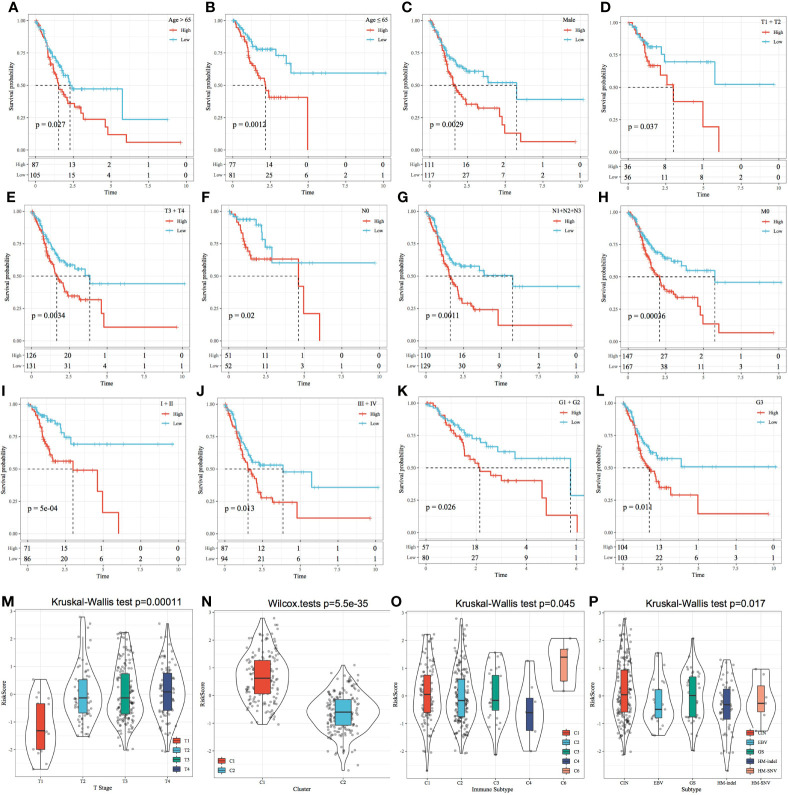
Risk score and prognostic analysis of clinical features. **(A–L)** Performance of the risk model based on different clinical features. **(M)** Comparison of the RiskScore among T Stage group samples. **(N)** Comparison of the RiskScore among the samples of molecular subtypes established in this study. **(O)** Comparison of RiskScore between samples with existing immune molecular subtype groups; **(P)** Comparison of RiskScore between samples with existing molecular subtype groups.

The relationship between the RiskScore and clinical features was analysed, and it was found that the RiskScore constructed based on the four-gene signature distinguished the high- and low-risk groups according to age, male sex, T stage, N stage, M0 stage, cancer stage and cancer grade (**Figures 11A–L**; p < 0.05). This finding consequently indicated that the risk model had a strong predictive ability across clinical features. Among the M stage samples, the M0 subgroup was divided into two groups based on the risk model; however, the M1 subgroup could not be divided based on the model. This inconsistency could be attributed to the relatively small M1 stage sample size. The T stage subgroups showed significant differences in terms of their RiskScores (**Figure 11M**; p < 0.001). The more advanced the T stage, the higher the RiskScore. Comparison of RiskScores between molecular subtypes showed that the RiskScores were significantly higher in the C1 subtype with a poorer prognosis than in the C2 subtype with a better prognosis (**Figure 11N**; p < 0.001). The RiskScores were significantly different between the available molecular subtypes (**Figures 11O–P**; p < 0.05).

### Relationship Between the RiskScore and Pathways

**Figure 12:**
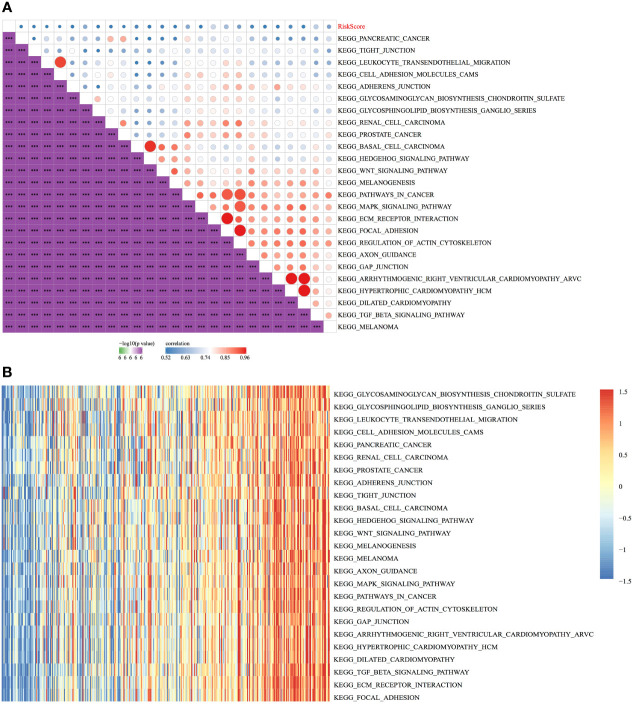
Relationship between the RiskScore and pathways. **(A)** Correlation between KEGG pathways with a correlation coefficient of >0.45 and the RiskScore. **(B)** Relationship between the ssGSEA scores of KEGG pathways with a correlation coefficient of >0.45 and increasing RiskScores in each sample, wherein the horizontal axis represents samples with increasing RiskScores from left to right. ***p < 0.001.

The relationship between the RiskScores and biological functions of different samples was analysed using GSEA. **Figure 12A** shows the functions with a correlation coefficient of >0.45. A total of 25 functions had a positive correlation with the RiskScores. Clustering analysis performed according to the enrichment scores of the top 25 most relevant KEGG pathways (**Figure 12B**) suggested that among the 25 pathways, the activity of KEGG_WNT_SIGNALING_PATHWAY, KEGG_ FOCAL_ADHESION, KEGG_PATHWAYS_IN_CANCER, KEGG_TGF_BETA_ SIGNALING_PATHWAY, and other tumour-related pathways increased with an increase in the RiskScore (**Figure 12**).

### Construction of a Nomogram

**Figure 13:**
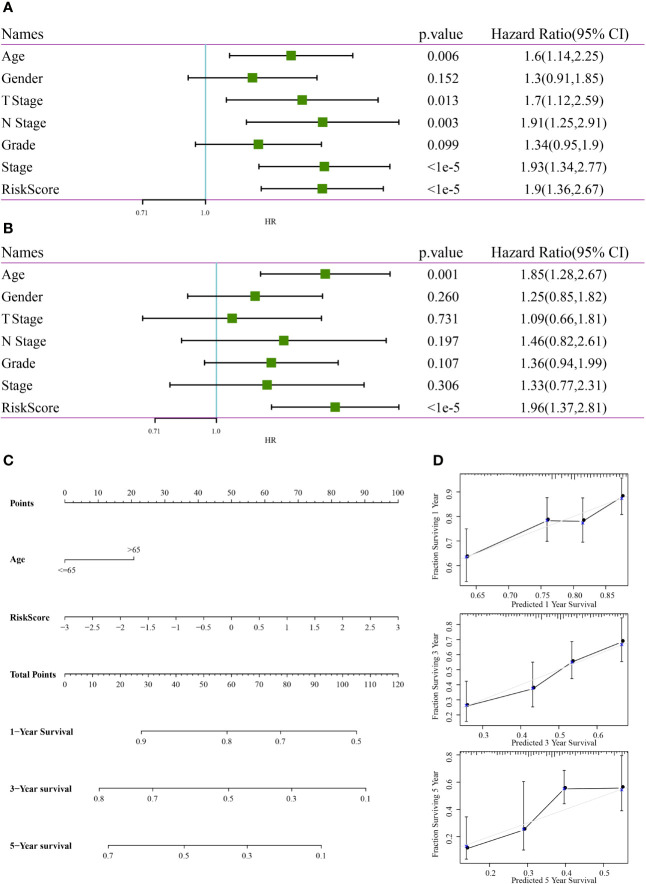
Construction of a nomogram. **(A)** Results of univariate analysis between clinical features and RiskScore. **(B)** Results of multivariate analysis between clinical features and RiskScore. **(C)** Nomogram constructed based on clinical features and RiskScore. **(D)** Calibration curve of the nomogram for predicting survival rates.

In TCGA dataset, univariate Cox regression analysis revealed that RiskScore was correlated with survival, whereas multivariate COX regression analysis revealed that RiskScore (HR = 1.96, 95% CI = 1.37–2.81, p < 1e-5) was correlated with survival. Age (HR = 1.85, 95% CI = 1.28–2.67, p < 0.001) was also correlated with patient prognosis (**Figures 13A, B**), consequently highlighting the good predictive performance of the four-gene signature in terms of clinical application value. Because the nomogram is an effective tool to visualise results, it is relatively more convenient for prognostic prediction (19). In this study, clinical features, such as age, and RiskScore were integrated into a nomogram based on the results of univariate and multivariate analyses (**Figure 13C**). The results demonstrated that RiskScore had the greatest impact on survival prediction, indicating that the four-gene signature was better in terms of predicting the prognosis. In addition, the nomogram data at 1, 3 and 5 years were corrected for visualising its performance (**Figure 13D**), which indicated that the risk model was accurate.

### Comparison of the Risk Model With Others

**Figure 14:**
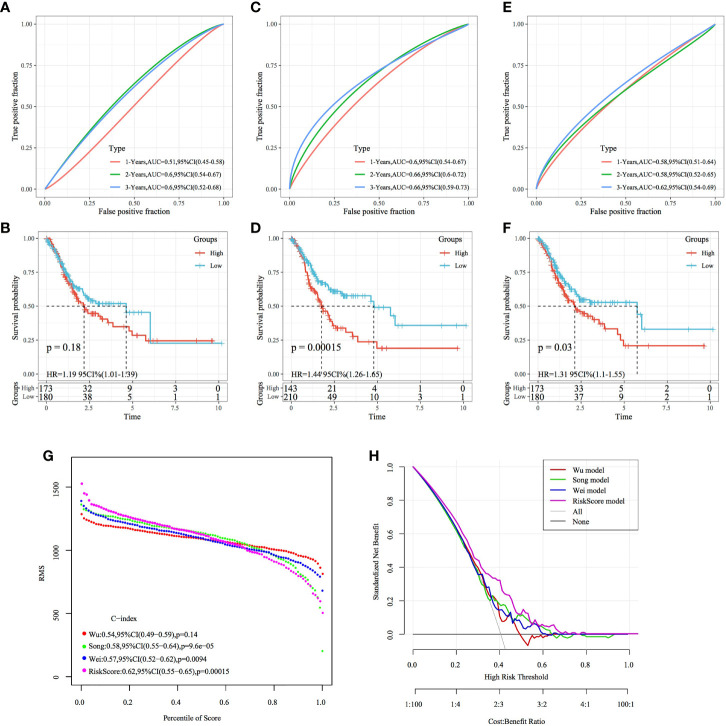
Comparison of the four-gene signature with other signatures. **(A, B)** ROC curve of a risk model based on a three-gene signature (Wu) and KM curve of the high- and low-risk groups. **(C, D)** ROC curve of a risk model based on a five-gene signature (Song) risk model and KM curve of the high- and low-risk groups. **(E, F)** ROC curve of a risk model based on an eight-gene signature (Wei) and KM curve of the high- and low-risk groups. **(G)** C-index curves of the models. **(H)** DCA curves of the models.

Based on the literature review, we selected the following three prognosis-related risk models and compared them with the four-gene signature established in this study: a three-gene signature (20), a five-gene signature (21) and an eight-gene signature (22). To compare the models, the RiskScores of each STAD sample in TCGA dataset were calculated according to the corresponding genes in these four models using the same method used to calculate the z-scores of the RiskScore, wherein samples with a RiskScore of >0 were classified as high risk, whereas those with a RiskScore of <0 were classified as low risk. Consequently, the intergroup prognostic differences were calculated. The ROC and KM curves (**Figures 14A–F**) showed that the AUC values of the three-, five- and eight-gene signature models at 1, 3 and 5 years were lower than the AUC value of the four-gene signature established in this study. Among them, the signatures of Song et al. and Wei et al. have significant prognostic difference in high and low groups, while the signature of Wu et al. has no significant difference in prognosis. The four-gene signature established in this study yielded a more valid model with fewer genes. To compare the predictive performance of these models for STAD samples, the concordance index (C-index) between the three models and the four-gene model of this study was calculated using the RMS package in R. The results showed that the C-index of the RiskScore model was the highest among the four models (**Figure 14G**). Therefore, the overall performance of the model was better than that of the other three models. The DCA curve revealed that the RiskScore has the highest net benefit when compared with the other models, thereby suggesting that the model established in this study has better clinical applicability (**Figure 14H**).

## Discussion

GC is a common malignant tumour of the digestive system. At present, the prognosis of patients with GC is established primarily based on tumour pathology (T), lymph node biopsy (N) and distant organ metastasis (M). However, owing to the genetic heterogeneity of GC, the prognosis of GC based on the TNM classification is often different. Moreover, at present, individualised and accurate prognosis prediction is not possible. Therefore, the identification of a more effective prognosis prediction method for GC is of paramount importance. In addition to being important biological features of GC, invasion and metastasis are key factors leading to tumour recurrence and affecting prognosis. Tumour spread is initiated after invasion of the basilar membrane by GC cells, which is one of the crucial steps leading to metastasis (23). GC cell invasion and metastasis involve an active process that is multistep, multistage, multigene, continuous, complex and multifactorially regulated. Invasion- and metastasis-related genes play an important role in this process. Potential prognostic biomarkers have been widely identified in several cancer types based on the comprehensive analysis of data from large public databases (24, 25). Multigene RiskScores constructed according to univariate and multivariate Cox regression models and lasso regression analysis have a higher prognostic value than single prognostic biomarkers (26–28). Therefore, in the present study, we used TCGA and GEO data to construct STAD molecular subtypes based on tumour invasion-related genes and constructed a four-gene signature to predict the prognosis of patients with GC based on DEGs between the two STAD molecular subtypes.

Based on the expression of tumour invasion-related genes, TCGA-STAD cohort was divided into two subtypes, with most genes being highly expressed in the C1 subtype. In addition, the C1 subtype had a worse prognosis than the C2 subtype, thus confirming the involvement of tumour invasion-related genes in the malignant progression of GC and their influence on prognosis. Inter-subtype DEG analysis showed that DEGs were mainly enriched in the following: ECM–receptor interaction, proteoglycans in cancer, focal adhesion, PI3K–Akt signalling pathway and other tumour-related pathways. In a study, the knockdown of OLFM4 enhanced the invasiveness of GC cells by activating focal adhesion signalling (29). ORAI2 promotes the occurrence and metastasis of GC through PI3K/Akt signalling and MAPK-dependent local adhesion dissociation (30), suggesting that STAD molecular subtypes may be involved in GC progression through invasion- and metastasis-related pathways. Comparison of molecular subtypes with different clinical features revealed a higher proportion of T2, T3 and T4 samples and a higher proportion of stage II, III and IV samples in the C1 subtype with a poor prognosis. Therefore, the molecular subtypes established in this study can stratify early and advance GC to some extent. In lung cancer, immune activation and escape reportedly precede tumour invasion (31). In this study, further assessment of the relationship between the molecular subtypes and immune scores showed that the immune scores of the C1 subtype were higher than those of the C2 subtype, regardless of the evaluation method. The proportion of immune subtype C3 (inflammation) was higher in the C1 subtype than in the C2 subtype when compared with the existing TCGA immunophenotyping. Systemic inflammation score (SIS) with preoperative serum albumin (Alb) levels and lymphocyte-to-monocyte ratio (LMR) has been proposed as a novel score for several malignancies, including GC (32). AEG-1-induced uncontrolled inflammation promotes GC presentation and predicts a poor prognosis (33). The results of this study indicate that invasion-related genes play an important role in the inflammatory response in GC; however, additional in-depth mechanistic studies are warranted to validate this finding.

Prognostically relevant DEGs were further screened to construct a four-gene signature, including the genes SERPINE1, MATN3, AMIGO2 and NOX4. SERPINE1 belongs to the serine protease inhibitor superfamily and is a multifunctional glycoprotein that plays a critical role in various cellular processes, such as EMT (34). SERPINE1 is overexpressed in the EMT subtype of GC (35); this overexpression consequently promotes EMT-mediated metastasis by activating STAT3 signalling in NSCLC cells (36). SERPINE1 expression induced by TGFβ stimulation increases the expression of EMT markers (37). The expression of SERPINE1 is higher in GC tissues than in healthy tissues, leading to a poor prognosis (38, 39). NADPH oxidase 4 (NOX4), a member of the NOX family, is an important source of reactive oxygen species and plays an important role in tumour cell proliferation and apoptosis. NOX4 can promote GC cell proliferation by activating the GLI1 pathway (40) and can regulate resistance to apoptosis in GC cells by generating reactive oxygen species and inducing EGFR (41). NOX4 knockdown inhibits the malignant progression of GC by inhibiting the JAK2/STAT3 pathway (42). Therefore, NOX4 can be used as a prognostic marker for GC (43). MATN3, a protein-coding gene, encodes a member of the protein family containing the von Willebrand factor A structural domain (44). MATN3 proteins are present in the extracellular matrix of the cartilage and play a role in homeostasis and cartilage and bone development (45). Studies on the mechanisms of MATN3 and AMIGO2 in GC are limited. Some studies have shown that MATN3 and AMIGO2 are overexpressed in gastric adenocarcinoma and can serve as markers of poor prognosis (46, 47). AMIGO2 plays a pathological role in tumour growth, collagen adhesion and migration of GC cells (48). In the future, we aim to perform an in-depth study on mechanisms underlying the involvement of MATN3 and AMIGO2 in GC. In this study, the RiskScore constructed using the four-gene signature could classify GC samples as high and low risk, and the prognosis of the high-risk group was worse than that of the low-risk group. Both internal and external datasets verified the robustness of the risk model. Furthermore, comparison of the RiskScores between molecular subtypes showed that the RiskScores of the C1 subtype with a poorer prognosis were significantly higher than those of the C2 subtype with a better prognosis, which is consistent with the previous findings of this study. Compared with three previously reported prognostic models for GC (20–22), the model established in this study incorporated fewer genes, was more operational in clinical practice and had the highest C-index value, indicating that its overall performance was better than that of the other three models. To the best of our knowledge, this study is the first to construct a prognostic model using tumour invasion-related genes, which can provide more insights into the role of prognostic models in the development of GC. The nomogram constructed based on the RiskScores can be used to guide prognosis prediction and clinical treatment of patients.

However, this study has several limitations. First, TCGA cohort is predominantly composed of patients with Caucasian and African ethnicities and lacks Asian representation in the data. Although a GEO external dataset was used for validation to reduce racial bias, further validation in real-world data with large sample size is necessary. Second, owing to the retrospective nature of the study, a prospective study is required for further validation. Finally, additional examination of the four genes identified is necessary to further examine their mechanism of action in the malignant progression of GC.

## Conclusion

In the present study, molecular typing of GC was performed based on tumour invasion-related genes. The four-gene signature developed for prognostic prediction using molecularly typed DEGs can be used as a tool to assess the prognostic risk of patients with GC.

## Data Availability Statement

The datasets presented in this study can be found in online repositories. The names of the repository/repositories and accession number(s) can be found in the article/**Supplementary Material**.

## Author Contributions

HG, HT, and YZ designed the study, performed data analysis and wrote the manuscript. QZ performed data collection; XH and LR supervised the manuscript. The current manuscript has been read and approved by all named authors.

## Conflict of Interest

The authors declare that the research was conducted in the absence of any commercial or financial relationships that could be construed as a potential conflict of interest.

## Publisher’s Note

All claims expressed in this article are solely those of the authors and do not necessarily represent those of their affiliated organizations, or those of the publisher, the editors and the reviewers. Any product that may be evaluated in this article, or claim that may be made by its manufacturer, is not guaranteed or endorsed by the publisher.

## Supplementary Material

Supplementary Figure 1 Comparison of the expression of four genes between tumour and healthy samples included in TCGA dataset.

Supplementary Figure 2 Comparison of the expression of four genes based on the TNM classification.
