# Supplementary material for: Molecular Typing of Gastric Cancer Based on Invasion-Related Genes and Prognosis-Related Features
Source: Front Oncol. 2022 Jun 3;12:848163. doi: 10.3389/fonc.2022.848163 (PMC9203697; doi:10.3389/fonc.2022.848163)
Supplement: Supplementary file 1 [file Table_1.docx]

Table S1. The 97 invasion-related gene set was obtained from CancerSEA

| No. | Name |
| --- | --- |
| 1 | AEBP1 |
| 2 | AKR1B1 |
| 3 | AMD1 |
| 4 | SLC25A5 |
| 5 | ATP5PB |
| 6 | BAG1 |
| 7 | BGN |
| 8 | C1QB |
| 9 | CALD1 |
| 10 | CAPG |
| 11 | CCNE1 |
| 12 | CDH11 |
| 13 | CKS1B |
| 14 | CKS2 |
| 15 | COL1A1 |
| 16 | COL1A2 |
| 17 | COL3A1 |
| 18 | COL5A1 |
| 19 | COL5A2 |
| 20 | COL6A2 |
| 21 | COL6A3 |
| 22 | COL10A1 |
| 23 | COL11A1 |
| 24 | COMP |
| 25 | CSE1L |
| 26 | VCAN |
| 27 | CTSK |
| 28 | DAB2 |
| 29 | DDX5 |
| 30 | EDNRA |
| 31 | FAP |
| 32 | FBN1 |
| 33 | FN1 |
| 34 | GNAS |
| 35 | H2AFZ |
| 36 | HMGB2 |
| 37 | HNRNPU |
| 38 | HSD17B4 |
| 39 | CCN1 |
| 40 | INHBA |
| 41 | LAMB1 |
| 42 | LAMC1 |
| 43 | LGALS1 |
| 44 | LOX |
| 45 | LOXL2 |
| 46 | LUM |
| 47 | MMP2 |
| 48 | MMP11 |
| 49 | HNRNPM |
| 50 | NDUFB7 |
| 51 | YBX1 |
| 52 | PDGFRB |
| 53 | PLAU |
| 54 | PRRX1 |
| 55 | PNN |
| 56 | PPIC |
| 57 | PROS1 |
| 58 | PSMA2 |
| 59 | PSMB4 |
| 60 | RGS4 |
| 61 | SNAI2 |
| 62 | SPOCK1 |
| 63 | TGFBI |
| 64 | THBS2 |
| 65 | THY1 |
| 66 | TNFAIP6 |
| 67 | UBE2V2 |
| 68 | ADAM12 |
| 69 | MFAP5 |
| 70 | ITGBL1 |
| 71 | TP53I3 |
| 72 | NUAK1 |
| 73 | HNRNPDL |
| 74 | TXNDC9 |
| 75 | LRRC17 |
| 76 | IFI30 |
| 77 | POSTN |
| 78 | CBX1 |
| 79 | NID2 |
| 80 | RRAS2 |
| 81 | RALY |
| 82 | SEPHS2 |
| 83 | HEY1 |
| 84 | MXRA5 |
| 85 | OLFML2B |
| 86 | TMEM158 |
| 87 | WWTR1 |
| 88 | GREM1 |
| 89 | NOX4 |
| 90 | CLEC4A |
| 91 | COPZ2 |
| 92 | ASPN |
| 93 | CEMIP |
| 94 | CRISPLD2 |
| 95 | TUBB6 |
| 96 | LRRC15 |
| 97 | TUBB |
