# Supplementary material for: Molecular Typing of Gastric Cancer Based on Invasion-Related Genes and Prognosis-Related Features
Source: Front Oncol. 2022 Jun 3;12:848163. doi: 10.3389/fonc.2022.848163 (PMC9203697; doi:10.3389/fonc.2022.848163)
Supplement: Supplementary file 2 [file Table_2.docx]

Table S2. A total of 569 DEGs were observed between the C1 and C2 subtypes

| Name | logFC | AveExpr | t | P.Value | adj.P.Val | B |
| --- | --- | --- | --- | --- | --- | --- |
| SFRP2 | 3.99251452 | 5.94319041 | 20.6532933 | 7.40E-63 | 5.76E-60 | 132.442957 |
| SFRP4 | 3.52112219 | 4.82241755 | 20.9813224 | 3.38E-64 | 3.49E-61 | 135.509611 |
| COL10A1 | 3.11070855 | 3.86291108 | 20.1036116 | 1.32E-60 | 7.36E-58 | 127.29382 |
| THBS4 | 2.94308099 | 3.29600939 | 14.8086853 | 5.38E-39 | 4.65E-37 | 77.851231 |
| FNDC1 | 2.85861843 | 3.97021509 | 21.613879 | 8.93E-67 | 1.20E-63 | 141.407294 |
| THBS2 | 2.68287833 | 5.45315359 | 21.8363307 | 1.11E-67 | 1.66E-64 | 143.475691 |
| GREM1 | 2.54197507 | 5.0798755 | 16.723362 | 9.68E-47 | 1.62E-44 | 95.5684494 |
| CCDC80 | 2.54142511 | 4.1185292 | 19.6693982 | 7.96E-59 | 3.68E-56 | 123.219139 |
| CNN1 | 2.53165686 | 5.50993579 | 10.9153014 | 4.04E-24 | 1.18E-22 | 43.8690675 |
| SPOCK1 | 2.52769229 | 3.22127843 | 21.4861175 | 2.96E-66 | 3.60E-63 | 140.217952 |
| ISLR | 2.51962994 | 5.83198936 | 20.8142534 | 1.63E-63 | 1.56E-60 | 133.948366 |
| DES | 2.45588384 | 6.49926054 | 7.22829155 | 3.01E-12 | 3.23E-11 | 16.8895531 |
| PRELP | 2.43709571 | 3.82626737 | 14.4561479 | 1.37E-37 | 1.06E-35 | 74.6375105 |
| COL8A1 | 2.41703468 | 3.87867411 | 22.2729289 | 1.89E-69 | 3.61E-66 | 147.525705 |
| HSPB6 | 2.41625064 | 5.45459625 | 9.83870382 | 2.31E-20 | 5.08E-19 | 35.3068613 |
| ASPN | 2.40839337 | 5.32854795 | 19.3646285 | 1.42E-57 | 5.77E-55 | 120.356452 |
| GAS1 | 2.38974712 | 3.21015727 | 20.1231798 | 1.10E-60 | 6.39E-58 | 127.477316 |
| MYH11 | 2.36954837 | 5.60342722 | 8.99610214 | 1.42E-17 | 2.52E-16 | 28.963585 |
| SULF1 | 2.35266073 | 5.49194178 | 19.901328 | 8.90E-60 | 4.59E-57 | 125.396241 |
| OGN | 2.33555071 | 3.12395702 | 11.434801 | 5.31E-26 | 1.83E-24 | 48.1600662 |
| MFAP5 | 2.33400612 | 3.25392252 | 20.2469907 | 3.41E-61 | 2.08E-58 | 128.638023 |
| TAGLN | 2.29365534 | 7.03023794 | 14.8486977 | 3.72E-39 | 3.26E-37 | 78.2171549 |
| FN1 | 2.25329973 | 7.28805732 | 16.9055215 | 1.75E-47 | 3.08E-45 | 97.2706365 |
| FBLN2 | 2.24078786 | 4.56227344 | 18.9623363 | 6.38E-56 | 2.35E-53 | 116.575606 |
| ACTG2 | 2.23016668 | 6.39175092 | 8.52209969 | 4.51E-16 | 6.89E-15 | 25.5518327 |
| FLNC | 2.21226332 | 4.25133359 | 10.6615895 | 3.23E-23 | 8.86E-22 | 41.8092742 |
| AEBP1 | 2.1939568 | 6.96672871 | 22.2784476 | 1.79E-69 | 3.61E-66 | 147.576813 |
| COMP | 2.18499743 | 2.81777034 | 11.8212003 | 2.00E-27 | 7.53E-26 | 51.4110492 |
| COL3A1 | 2.17460517 | 9.23921712 | 20.7882517 | 2.08E-63 | 1.86E-60 | 133.705262 |
| MGP | 2.16599372 | 6.82433954 | 13.9979955 | 8.91E-36 | 6.03E-34 | 70.4912903 |
| SPON1 | 2.15558231 | 4.5207673 | 13.816396 | 4.61E-35 | 2.96E-33 | 68.8582763 |
| C7 | 2.14210438 | 3.1007176 | 9.76968233 | 3.95E-20 | 8.54E-19 | 34.7746876 |
| COL1A2 | 2.14070325 | 8.4230212 | 21.0580298 | 1.64E-64 | 1.84E-61 | 136.225967 |
| PTGIS | 2.13322595 | 3.43175999 | 12.8028682 | 3.95E-31 | 1.89E-29 | 59.8708119 |
| LMOD1 | 2.12204596 | 4.3540481 | 11.0413165 | 1.42E-24 | 4.36E-23 | 44.9010926 |
| COL1A1 | 2.11190861 | 9.58062483 | 17.7502451 | 6.06E-51 | 1.31E-48 | 105.185849 |
| PLN | 2.10061412 | 3.45765685 | 11.6175998 | 1.13E-26 | 4.09E-25 | 49.6920254 |
| PODN | 2.09081283 | 4.34935619 | 16.1965046 | 1.36E-44 | 1.80E-42 | 90.6580437 |
| DPYSL3 | 2.08343541 | 5.33772507 | 15.6827903 | 1.64E-42 | 1.87E-40 | 85.8926513 |
| HAND2 | 2.08281064 | 2.03891257 | 11.6558328 | 8.18E-27 | 2.98E-25 | 50.0138233 |
| CHRDL2 | 2.08028503 | 3.76683187 | 10.416854 | 2.35E-22 | 6.02E-21 | 39.8460731 |
| COL11A1 | 2.07713351 | 2.31248544 | 13.7639802 | 7.41E-35 | 4.60E-33 | 68.3881083 |
| CYP1B1 | 2.07287131 | 3.10803051 | 13.9892361 | 9.64E-36 | 6.46E-34 | 70.4123803 |
| FBLN1 | 2.05812153 | 5.32600994 | 12.8747896 | 2.09E-31 | 1.01E-29 | 60.5007955 |
| HSPB7 | 2.05762862 | 2.99683699 | 10.6112872 | 4.87E-23 | 1.31E-21 | 41.4038268 |
| MYL9 | 2.05345423 | 7.78882231 | 13.2155754 | 1.02E-32 | 5.49E-31 | 63.5028067 |
| LUM | 2.04876829 | 8.21910777 | 21.9388105 | 4.27E-68 | 7.15E-65 | 144.427491 |
| ANTXR1 | 2.03885034 | 5.35971196 | 22.2875388 | 1.65E-69 | 3.61E-66 | 147.661001 |
| SYNPO2 | 2.03326578 | 3.9064694 | 9.17867697 | 3.63E-18 | 6.80E-17 | 30.3087977 |
| MFAP4 | 2.02883509 | 6.55010055 | 12.7021644 | 9.59E-31 | 4.48E-29 | 58.9908964 |
| OMD | 2.02677097 | 2.12253203 | 15.6581309 | 2.06E-42 | 2.25E-40 | 85.6645559 |
| PRRX1 | 2.0240942 | 3.15321925 | 22.4720703 | 2.95E-70 | 9.89E-67 | 149.368516 |
| BGN | 2.02293065 | 8.7720435 | 20.6486242 | 7.74E-63 | 5.76E-60 | 132.399271 |
| CTHRC1 | 2.00975822 | 5.44951035 | 17.6290301 | 1.90E-50 | 3.93E-48 | 104.048299 |
| MXRA8 | 1.99704927 | 5.88768147 | 18.8440146 | 1.95E-55 | 6.55E-53 | 115.46334 |
| FBN1 | 1.99656696 | 4.30792347 | 20.7003583 | 4.75E-63 | 3.98E-60 | 132.883264 |
| PDLIM3 | 1.99359155 | 3.97596946 | 15.9340117 | 1.58E-43 | 1.94E-41 | 88.219946 |
| SFRP1 | 1.98521154 | 2.2432909 | 11.5707982 | 1.68E-26 | 6.05E-25 | 49.2987532 |
| MMP2 | 1.9837649 | 7.05907849 | 18.6030758 | 1.91E-54 | 5.44E-52 | 113.198368 |
| MRGPRF | 1.98273286 | 3.69571431 | 13.8883926 | 2.40E-35 | 1.57E-33 | 69.5049503 |
| SSC5D | 1.96863832 | 2.99048719 | 17.1054725 | 2.66E-48 | 4.94E-46 | 99.1412726 |
| ACTA2 | 1.94521421 | 7.66539784 | 14.7497563 | 9.26E-39 | 7.71E-37 | 77.3127309 |
| MYLK | 1.94377915 | 4.26529282 | 12.2003946 | 7.65E-29 | 3.19E-27 | 54.6465822 |
| COL14A1 | 1.93792991 | 3.88489188 | 14.1756394 | 1.77E-36 | 1.24E-34 | 72.0946208 |
| AOC3 | 1.92825758 | 4.23509437 | 14.2331791 | 1.05E-36 | 7.48E-35 | 72.6151463 |
| ITGBL1 | 1.90782155 | 1.99450208 | 17.8672776 | 2.01E-51 | 4.56E-49 | 106.284558 |
| BARX1 | 1.90629332 | 4.355037 | 10.4550449 | 1.73E-22 | 4.47E-21 | 40.1508539 |
| COL5A1 | 1.90385791 | 6.18580159 | 19.3745612 | 1.29E-57 | 5.41E-55 | 120.449776 |
| POSTN | 1.90256872 | 6.4407696 | 15.9620914 | 1.21E-43 | 1.52E-41 | 88.4804575 |
| SERPINF1 | 1.8966448 | 5.75212718 | 18.4372741 | 9.16E-54 | 2.56E-51 | 111.639843 |
| INHBA | 1.89586518 | 4.14243214 | 18.828521 | 2.26E-55 | 7.39E-53 | 115.317691 |
| DCN | 1.89478396 | 5.65295692 | 18.068737 | 2.99E-52 | 7.15E-50 | 108.176666 |
| HTRA3 | 1.88634736 | 5.27616505 | 18.2532649 | 5.22E-53 | 1.37E-50 | 109.910454 |
| ADAMTS2 | 1.88239063 | 4.30858278 | 17.085389 | 3.21E-48 | 5.81E-46 | 98.9532855 |
| COL6A3 | 1.88034544 | 6.31258547 | 18.8110457 | 2.67E-55 | 8.52E-53 | 115.153413 |
| EFEMP1 | 1.88025092 | 4.91815214 | 13.7775943 | 6.55E-35 | 4.10E-33 | 68.5101745 |
| CILP | 1.87837848 | 2.01282745 | 12.6580764 | 1.41E-30 | 6.51E-29 | 58.6064851 |
| SYNM | 1.87601304 | 3.64349571 | 8.25466646 | 3.01E-15 | 4.28E-14 | 23.680668 |
| THBS1 | 1.87581116 | 6.50422535 | 16.7839048 | 5.48E-47 | 9.29E-45 | 96.1339647 |
| ISM1 | 1.87320962 | 2.13736136 | 16.0493803 | 5.37E-44 | 6.85E-42 | 89.2907558 |
| VCAN | 1.87080212 | 4.87070025 | 18.8966256 | 1.19E-55 | 4.19E-53 | 115.957909 |
| ITGA11 | 1.86688235 | 3.20763857 | 20.2483629 | 3.36E-61 | 2.08E-58 | 128.650884 |
| MSRB3 | 1.8640427 | 3.66346034 | 15.4689741 | 1.20E-41 | 1.26E-39 | 83.9170844 |
| MEDAG | 1.85348928 | 2.98406863 | 17.6565116 | 1.47E-50 | 3.12E-48 | 104.306161 |
| CDH11 | 1.8444702 | 3.84212747 | 23.0449035 | 1.44E-72 | 9.67E-69 | 154.652452 |
| FAP | 1.83403481 | 2.41798703 | 23.0691476 | 1.15E-72 | 9.67E-69 | 154.875505 |
| SCARF2 | 1.82968364 | 4.13993076 | 19.3099371 | 2.38E-57 | 9.39E-55 | 119.842563 |
| DDR2 | 1.82339514 | 3.45603289 | 17.3164371 | 3.64E-49 | 6.96E-47 | 101.117136 |
| CPXM2 | 1.82112593 | 3.25852149 | 12.4500086 | 8.73E-30 | 3.81E-28 | 56.7991883 |
| OLFML2B | 1.81765138 | 4.72270547 | 16.010277 | 7.74E-44 | 9.78E-42 | 88.9276755 |
| GUCY1A1 | 1.81581024 | 3.71601859 | 15.8442003 | 3.65E-43 | 4.40E-41 | 87.3872193 |
| CLMP | 1.80612847 | 3.18745929 | 15.6787541 | 1.70E-42 | 1.92E-40 | 85.8553129 |
| TIMP3 | 1.80373059 | 3.25014672 | 16.6280892 | 2.37E-46 | 3.83E-44 | 94.6790088 |
| MMP11 | 1.79725951 | 4.76408984 | 10.8071597 | 9.83E-24 | 2.79E-22 | 42.9881162 |
| PLXDC2 | 1.79012551 | 3.73069241 | 19.491953 | 4.26E-58 | 1.90E-55 | 121.552623 |
| RSPO3 | 1.78237801 | 2.92773162 | 12.772639 | 5.16E-31 | 2.43E-29 | 59.6064098 |
| TNS1 | 1.77971373 | 5.19895826 | 12.5181241 | 4.82E-30 | 2.14E-28 | 57.3895787 |
| CHRDL1 | 1.77790585 | 2.1515767 | 10.6584688 | 3.31E-23 | 9.07E-22 | 41.7840923 |
| SMOC2 | 1.77205425 | 4.74443293 | 10.6875757 | 2.61E-23 | 7.27E-22 | 42.0191149 |
| CALD1 | 1.77050022 | 6.70705798 | 14.9057139 | 2.20E-39 | 1.99E-37 | 78.7389764 |
| LRRC15 | 1.76627396 | 2.36135722 | 13.6409529 | 2.25E-34 | 1.36E-32 | 67.286693 |
| ANGPTL2 | 1.76325477 | 5.46601557 | 17.5497377 | 4.02E-50 | 8.05E-48 | 103.304433 |
| EMILIN1 | 1.76149838 | 6.01870918 | 16.6244346 | 2.45E-46 | 3.91E-44 | 94.6449031 |
| COL12A1 | 1.74520249 | 5.57826848 | 16.161038 | 1.89E-44 | 2.46E-42 | 90.3282656 |
| GFPT2 | 1.72608965 | 2.46979644 | 20.3060454 | 1.95E-61 | 1.38E-58 | 129.191458 |
| TNC | 1.71744285 | 5.12125465 | 11.0101007 | 1.84E-24 | 5.61E-23 | 44.6449027 |
| SPARC | 1.71482987 | 8.73614753 | 19.7862099 | 2.64E-59 | 1.26E-56 | 124.315813 |
| CCL21 | 1.71419848 | 5.51809996 | 8.07378664 | 1.06E-14 | 1.44E-13 | 22.4380389 |
| COL6A2 | 1.7128777 | 7.90141449 | 18.1979321 | 8.81E-53 | 2.23E-50 | 109.390499 |
| COL8A2 | 1.71286984 | 2.87193615 | 18.8597164 | 1.68E-55 | 5.79E-53 | 115.610946 |
| FIBIN | 1.71160338 | 3.12048452 | 18.6811656 | 9.12E-55 | 2.84E-52 | 113.932452 |
| FLNA | 1.69825285 | 8.17458469 | 11.9784054 | 5.19E-28 | 2.04E-26 | 52.7471984 |
| DPT | 1.69402937 | 2.83786202 | 11.2206067 | 3.20E-25 | 1.04E-23 | 46.3793193 |
| COL5A2 | 1.68797841 | 6.059451 | 17.8953993 | 1.54E-51 | 3.55E-49 | 106.548621 |
| TIMP2 | 1.68479608 | 6.90302483 | 19.8720607 | 1.17E-59 | 5.83E-57 | 125.121591 |
| FGF7 | 1.68199324 | 2.62827362 | 14.5829066 | 4.28E-38 | 3.48E-36 | 75.7908429 |
| SRPX | 1.67886129 | 3.23119465 | 13.770076 | 7.01E-35 | 4.37E-33 | 68.4427598 |
| SPARCL1 | 1.67481508 | 6.87629758 | 11.8352815 | 1.77E-27 | 6.69E-26 | 51.5304208 |
| RGMA | 1.66912359 | 2.45669327 | 10.6307171 | 4.16E-23 | 1.13E-21 | 41.560319 |
| BOC | 1.66806531 | 2.19197834 | 14.2437908 | 9.52E-37 | 6.86E-35 | 72.7112062 |
| IGF2 | 1.66334674 | 4.71808511 | 7.6289221 | 2.19E-13 | 2.61E-12 | 19.4638191 |
| AKAP12 | 1.66320496 | 3.65093962 | 12.3094431 | 2.97E-29 | 1.27E-27 | 55.5848457 |
| CPXM1 | 1.65492174 | 3.88871038 | 14.6205939 | 3.03E-38 | 2.48E-36 | 76.1342311 |
| LTBP2 | 1.65366086 | 4.93090013 | 15.7203986 | 1.16E-42 | 1.34E-40 | 86.2406447 |
| GXYLT2 | 1.65202657 | 2.50835655 | 16.2010383 | 1.30E-44 | 1.74E-42 | 90.7002065 |
| MRC2 | 1.64854588 | 4.85910108 | 18.0925579 | 2.39E-52 | 5.81E-50 | 108.40045 |
| TMEM119 | 1.64806475 | 4.09033042 | 15.0503729 | 5.79E-40 | 5.51E-38 | 80.0649365 |
| ELN | 1.64761268 | 4.4694286 | 11.4082619 | 6.64E-26 | 2.25E-24 | 47.9385772 |
| CTSK | 1.6423624 | 6.32243085 | 18.6638241 | 1.07E-54 | 3.20E-52 | 113.769432 |
| IGFBP5 | 1.64082481 | 7.39036046 | 13.4032028 | 1.90E-33 | 1.06E-31 | 65.1670168 |
| C1S | 1.64055328 | 6.28735437 | 15.6647727 | 1.94E-42 | 2.17E-40 | 85.7259851 |
| CRYAB | 1.63927715 | 3.58620861 | 11.8496253 | 1.57E-27 | 5.93E-26 | 51.6520827 |
| FHL1 | 1.6302117 | 4.44749543 | 9.50419326 | 3.07E-19 | 6.17E-18 | 32.7480379 |
| SERPING1 | 1.62739048 | 7.39133504 | 14.973623 | 1.18E-39 | 1.09E-37 | 79.3610834 |
| LOX | 1.62604091 | 4.10161097 | 18.0205001 | 4.71E-52 | 1.11E-49 | 107.723544 |
| ECRG4 | 1.62197225 | 2.13116306 | 8.21830253 | 3.88E-15 | 5.47E-14 | 23.4293435 |
| BICC1 | 1.61979833 | 2.7664835 | 18.6733116 | 9.82E-55 | 2.99E-52 | 113.85862 |
| PCOLCE | 1.61734783 | 5.18724785 | 18.3677911 | 1.77E-53 | 4.83E-51 | 110.986771 |
| FSTL1 | 1.61306658 | 6.14054066 | 18.9604489 | 6.49E-56 | 2.35E-53 | 116.557864 |
| ZCCHC24 | 1.61050733 | 4.00829943 | 14.669689 | 1.93E-38 | 1.59E-36 | 76.5818879 |
| ROR2 | 1.60940687 | 2.66315791 | 14.2418688 | 9.69E-37 | 6.95E-35 | 72.693807 |
| SLIT2 | 1.6086315 | 1.74992056 | 15.7667635 | 7.51E-43 | 8.90E-41 | 86.6698661 |
| CST2 | 1.60333248 | 2.91305292 | 9.82364588 | 2.60E-20 | 5.70E-19 | 35.1905777 |
| APOD | 1.60331073 | 5.42273373 | 6.7208845 | 7.19E-11 | 6.72E-10 | 13.7789712 |
| C1R | 1.60059371 | 6.23381157 | 16.3347922 | 3.71E-45 | 5.29E-43 | 91.9448741 |
| MRVI1 | 1.59981846 | 3.87561154 | 12.5982023 | 2.39E-30 | 1.09E-28 | 58.0852403 |
| NNMT | 1.593793 | 5.17891593 | 14.8826908 | 2.72E-39 | 2.45E-37 | 78.5282097 |
| ANGPTL1 | 1.59253629 | 1.82275053 | 10.6625906 | 3.21E-23 | 8.80E-22 | 41.8173534 |
| THY1 | 1.59250679 | 5.52247436 | 17.8436201 | 2.51E-51 | 5.60E-49 | 106.06243 |
| PDGFRB | 1.59130874 | 5.87920834 | 17.4196265 | 1.37E-49 | 2.71E-47 | 102.084317 |
| RAB31 | 1.59091862 | 4.99965256 | 19.9421355 | 6.06E-60 | 3.25E-57 | 125.779148 |
| C3 | 1.58786062 | 7.09768672 | 9.10642889 | 6.24E-18 | 1.15E-16 | 29.7744654 |
| KCNMA1 | 1.5855891 | 2.03436738 | 10.4213155 | 2.27E-22 | 5.82E-21 | 39.8816471 |
| NDN | 1.58556002 | 4.00783491 | 15.5078631 | 8.35E-42 | 8.82E-40 | 84.2760244 |
| NEXN | 1.58321856 | 3.84987901 | 11.8786499 | 1.22E-27 | 4.66E-26 | 51.8984587 |
| TWIST2 | 1.58234675 | 2.29187484 | 16.5503738 | 4.91E-46 | 7.40E-44 | 93.9539337 |
| TGFB3 | 1.58122193 | 2.87393553 | 18.2097876 | 7.87E-53 | 2.03E-50 | 109.5019 |
| PRUNE2 | 1.57544624 | 2.71163291 | 8.46032334 | 7.01E-16 | 1.05E-14 | 25.1160675 |
| GPC6 | 1.57407699 | 3.1403315 | 16.5842508 | 3.58E-46 | 5.51E-44 | 94.2699509 |
| LRRC32 | 1.57066518 | 4.889212 | 16.5272828 | 6.10E-46 | 8.99E-44 | 93.7385789 |
| SYNDIG1 | 1.56686546 | 2.12926116 | 19.2928411 | 2.80E-57 | 1.07E-54 | 119.681916 |
| GLT8D2 | 1.56556787 | 3.006944 | 22.9740477 | 2.78E-72 | 1.24E-68 | 154.000282 |
| CLEC11A | 1.56522003 | 4.25397039 | 17.3795625 | 2.01E-49 | 3.89E-47 | 101.708749 |
| CAVIN1 | 1.56363801 | 6.7188424 | 15.6619999 | 1.99E-42 | 2.19E-40 | 85.7003392 |
| CYBRD1 | 1.56297254 | 5.17723175 | 12.6755703 | 1.21E-30 | 5.62E-29 | 58.7589578 |
| ADAMTS12 | 1.56286743 | 2.89272102 | 15.9582128 | 1.26E-43 | 1.56E-41 | 88.4444692 |
| PGM5 | 1.56221488 | 2.4671961 | 9.0079509 | 1.30E-17 | 2.32E-16 | 29.0503746 |
| AHNAK2 | 1.56094403 | 3.17315804 | 9.83912925 | 2.30E-20 | 5.07E-19 | 35.3101481 |
| MAP1A | 1.55899363 | 2.86737864 | 16.2244648 | 1.04E-44 | 1.41E-42 | 90.9180994 |
| DACT1 | 1.55134917 | 3.17908139 | 15.895682 | 2.26E-43 | 2.75E-41 | 87.8644596 |
| HSPB8 | 1.55048345 | 3.87714232 | 9.29031829 | 1.57E-18 | 2.99E-17 | 31.1395696 |
| JPH2 | 1.54846452 | 2.31089703 | 9.85140461 | 2.09E-20 | 4.62E-19 | 35.405021 |
| PLIN4 | 1.5476127 | 1.97388571 | 8.68382065 | 1.40E-16 | 2.24E-15 | 26.7024466 |
| ADAM12 | 1.54364198 | 2.36441649 | 14.448412 | 1.47E-37 | 1.13E-35 | 74.5672075 |
| CCN4 | 1.53816814 | 2.79990272 | 16.5237539 | 6.31E-46 | 9.19E-44 | 93.7056705 |
| DACT3 | 1.53629598 | 2.41429982 | 12.68344 | 1.13E-30 | 5.26E-29 | 58.827574 |
| TPM2 | 1.53428335 | 6.5152889 | 10.4252279 | 2.19E-22 | 5.65E-21 | 39.9128498 |
| GGT5 | 1.5306054 | 4.0373187 | 16.8403967 | 3.22E-47 | 5.53E-45 | 96.6618477 |
| MEOX2 | 1.52802557 | 1.54703894 | 14.1181953 | 2.99E-36 | 2.05E-34 | 71.5755382 |
| SGCD | 1.52431657 | 2.26925093 | 15.6636858 | 1.96E-42 | 2.17E-40 | 85.7159329 |
| MOXD1 | 1.52364343 | 2.8377031 | 13.8328924 | 3.97E-35 | 2.57E-33 | 69.0063587 |
| LAMA2 | 1.52107353 | 2.55668171 | 14.9831242 | 1.08E-39 | 1.00E-37 | 79.4481724 |
| MFAP2 | 1.5133172 | 4.00791257 | 11.9732047 | 5.43E-28 | 2.12E-26 | 52.7028753 |
| LTBP1 | 1.50969049 | 4.88273884 | 13.6491896 | 2.09E-34 | 1.26E-32 | 67.3603382 |
| TACR2 | 1.50925594 | 2.32177392 | 7.47311086 | 6.13E-13 | 7.00E-12 | 18.4506165 |
| SOD3 | 1.50853209 | 6.29336551 | 11.5158819 | 2.68E-26 | 9.45E-25 | 48.8382083 |
| F13A1 | 1.50760579 | 2.85686105 | 9.57551266 | 1.78E-19 | 3.68E-18 | 33.2892483 |
| CERCAM | 1.50705701 | 4.17911227 | 15.3582844 | 3.35E-41 | 3.40E-39 | 82.8963882 |
| TUBA1A | 1.50389505 | 6.08588806 | 15.2909367 | 6.26E-41 | 6.30E-39 | 82.2760703 |
| FERMT2 | 1.50070174 | 3.80257789 | 14.131229 | 2.65E-36 | 1.84E-34 | 71.6932633 |
| TAFA5 | 1.49960065 | 2.44825336 | 15.8231641 | 4.44E-43 | 5.31E-41 | 87.1922863 |
| CLIP3 | 1.49888673 | 3.89243823 | 13.4200388 | 1.63E-33 | 9.17E-32 | 65.3167256 |
| CACNA1H | 1.49743654 | 3.52588485 | 11.1847711 | 4.32E-25 | 1.39E-23 | 46.0829459 |
| EDNRA | 1.49707724 | 3.64441974 | 17.0944524 | 2.95E-48 | 5.41E-46 | 99.0381183 |
| STON1 | 1.49576617 | 2.21827346 | 15.1198763 | 3.05E-40 | 2.92E-38 | 80.7030116 |
| CRISPLD2 | 1.49551442 | 4.62594279 | 16.6343092 | 2.23E-46 | 3.65E-44 | 94.7370585 |
| CYS1 | 1.49538613 | 2.22315013 | 11.4548987 | 4.48E-26 | 1.56E-24 | 48.3279538 |
| SRPX2 | 1.49279422 | 3.44817336 | 17.6446971 | 1.64E-50 | 3.44E-48 | 104.195301 |
| KCNE4 | 1.49264067 | 2.49463058 | 16.469483 | 1.05E-45 | 1.51E-43 | 93.199687 |
| CPE | 1.49184762 | 4.16168072 | 11.1599569 | 5.31E-25 | 1.68E-23 | 45.8779884 |
| EFEMP2 | 1.4904551 | 4.02733127 | 18.2934405 | 3.57E-53 | 9.57E-51 | 110.288006 |
| PDGFRL | 1.49000516 | 2.1438919 | 15.2477874 | 9.33E-41 | 9.33E-39 | 81.8789262 |
| SYNC | 1.48906776 | 1.8196796 | 13.71122 | 1.19E-34 | 7.30E-33 | 67.9153967 |
| MN1 | 1.48897451 | 2.69597634 | 11.6974043 | 5.75E-27 | 2.12E-25 | 50.3642532 |
| IGFBP4 | 1.48856044 | 8.71281636 | 13.3335624 | 3.54E-33 | 1.95E-31 | 64.5484153 |
| ST6GALNAC5 | 1.4836257 | 2.0396054 | 18.6315444 | 1.46E-54 | 4.25E-52 | 113.465985 |
| OLFML3 | 1.47133189 | 4.69127627 | 13.0631792 | 3.94E-32 | 2.02E-30 | 62.1569463 |
| CCDC8 | 1.47068974 | 2.13980339 | 13.4402741 | 1.36E-33 | 7.74E-32 | 65.4967427 |
| CCN2 | 1.46952411 | 7.635211 | 14.3259319 | 4.50E-37 | 3.37E-35 | 73.4554208 |
| RGS4 | 1.46806893 | 1.97063972 | 14.8015086 | 5.75E-39 | 4.94E-37 | 77.7856223 |
| CCN1 | 1.46208855 | 6.34629814 | 11.9272988 | 8.06E-28 | 3.12E-26 | 52.311995 |
| RBPMS2 | 1.45261715 | 3.37672619 | 8.71462175 | 1.12E-16 | 1.81E-15 | 26.9231868 |
| INMT | 1.45003219 | 2.46812933 | 12.7900219 | 4.42E-31 | 2.11E-29 | 59.7584223 |
| PALLD | 1.44802721 | 5.57988537 | 13.4200769 | 1.63E-33 | 9.17E-32 | 65.3170648 |
| EVC | 1.44789226 | 2.74233372 | 14.7181897 | 1.24E-38 | 1.02E-36 | 77.0244812 |
| FBLN5 | 1.44225676 | 4.32468953 | 13.1466535 | 1.88E-32 | 9.95E-31 | 62.8934706 |
| VSTM4 | 1.44197042 | 2.22370036 | 18.1439304 | 1.47E-52 | 3.64E-50 | 108.883099 |
| COPZ2 | 1.44130042 | 3.19516519 | 16.8618706 | 2.63E-47 | 4.58E-45 | 96.8625582 |
| ARHGEF25 | 1.43464649 | 2.66729479 | 13.0816662 | 3.35E-32 | 1.73E-30 | 62.319923 |
| TGFB1I1 | 1.4345247 | 4.58692027 | 14.4438897 | 1.53E-37 | 1.17E-35 | 74.5261134 |
| ACKR1 | 1.43439904 | 3.62987528 | 7.89795112 | 3.56E-14 | 4.60E-13 | 21.2483138 |
| ABCC9 | 1.43396301 | 2.02235307 | 13.5432109 | 5.41E-34 | 3.17E-32 | 66.4138353 |
| NTM | 1.42743315 | 1.74859466 | 19.4706437 | 5.21E-58 | 2.25E-55 | 121.35245 |
| FBXO32 | 1.42727579 | 4.38315195 | 11.9816694 | 5.05E-28 | 1.99E-26 | 52.7750197 |
| ADGRA2 | 1.42324868 | 4.4856056 | 14.4580023 | 1.35E-37 | 1.05E-35 | 74.6543649 |
| TMEM47 | 1.42271826 | 4.09463838 | 13.7179404 | 1.12E-34 | 6.90E-33 | 67.9755775 |
| C11orf96 | 1.422592 | 5.71587266 | 12.5095056 | 5.19E-30 | 2.30E-28 | 57.3148083 |
| NRP2 | 1.41941894 | 3.43425006 | 14.1324518 | 2.62E-36 | 1.83E-34 | 71.70431 |
| GPNMB | 1.41237017 | 5.55784082 | 10.7417916 | 1.68E-23 | 4.71E-22 | 42.4577531 |
| COL6A1 | 1.41028595 | 7.64878176 | 14.2472169 | 9.23E-37 | 6.69E-35 | 72.7422244 |
| GUCY1B1 | 1.4082089 | 3.9488912 | 15.1415695 | 2.49E-40 | 2.46E-38 | 80.9022951 |
| FXYD6 | 1.40647476 | 2.77995332 | 12.5925975 | 2.51E-30 | 1.14E-28 | 58.0364949 |
| FGFR1 | 1.40358841 | 3.3597703 | 13.1675162 | 1.56E-32 | 8.33E-31 | 63.0778021 |
| AXL | 1.4021058 | 4.81977544 | 15.1215541 | 3.00E-40 | 2.89E-38 | 80.7184225 |
| LHFPL6 | 1.40137513 | 4.79633427 | 14.7675439 | 7.86E-39 | 6.63E-37 | 77.4752225 |
| IGFBP6 | 1.39639243 | 4.73865857 | 13.0924153 | 3.04E-32 | 1.59E-30 | 62.414722 |
| SLC24A3 | 1.39366008 | 3.08790461 | 13.5108198 | 7.24E-34 | 4.18E-32 | 66.125013 |
| WNT2 | 1.39268934 | 2.34433632 | 11.2537432 | 2.43E-25 | 7.96E-24 | 46.6537715 |
| FSTL3 | 1.39225206 | 4.62455169 | 11.3025669 | 1.61E-25 | 5.41E-24 | 47.058848 |
| MXRA5 | 1.3886182 | 5.85395968 | 11.1604423 | 5.29E-25 | 1.67E-23 | 45.8819952 |
| KCNMB1 | 1.38759556 | 2.58641219 | 9.5544672 | 2.09E-19 | 4.28E-18 | 33.1292953 |
| GEM | 1.38643715 | 5.14168789 | 13.110469 | 2.59E-32 | 1.36E-30 | 62.574002 |
| SSPN | 1.38322575 | 3.15239012 | 13.5634785 | 4.51E-34 | 2.66E-32 | 66.5946674 |
| HOPX | 1.38242492 | 2.89168076 | 11.7840476 | 2.75E-27 | 1.03E-25 | 51.096387 |
| LIMS2 | 1.37895121 | 2.98074694 | 11.6794272 | 6.69E-27 | 2.45E-25 | 50.2126458 |
| PI16 | 1.37576238 | 1.81724034 | 8.35815509 | 1.45E-15 | 2.12E-14 | 24.4000144 |
| SORBS1 | 1.37547094 | 4.22397587 | 8.28449735 | 2.44E-15 | 3.50E-14 | 23.8874013 |
| FRMD6 | 1.37484782 | 2.70982784 | 16.2363969 | 9.33E-45 | 1.29E-42 | 91.0290997 |
| RNF150 | 1.37386834 | 1.82319257 | 12.0393174 | 3.08E-28 | 1.23E-26 | 53.2669266 |
| SERPINE1 | 1.36794575 | 5.84429227 | 9.33404998 | 1.12E-18 | 2.17E-17 | 31.4666631 |
| SHISA3 | 1.36446972 | 1.80913194 | 9.2037302 | 3.01E-18 | 5.65E-17 | 30.494694 |
| CST1 | 1.362371 | 6.0721315 | 5.20070043 | 3.36E-07 | 2.05E-06 | 5.55618563 |
| PODNL1 | 1.36162141 | 2.30166077 | 16.9537094 | 1.11E-47 | 1.98E-45 | 97.7212569 |
| COL16A1 | 1.36155603 | 3.75196167 | 14.4021061 | 2.24E-37 | 1.71E-35 | 74.1465853 |
| TWIST1 | 1.3612514 | 2.30651962 | 13.4726877 | 1.02E-33 | 5.81E-32 | 65.7852826 |
| ANOS1 | 1.36073038 | 2.1892693 | 15.7458186 | 9.12E-43 | 1.06E-40 | 86.4759427 |
| CD248 | 1.36055953 | 5.35077129 | 14.557176 | 5.42E-38 | 4.35E-36 | 75.5565242 |
| TSPAN2 | 1.35957949 | 3.05881882 | 10.6016964 | 5.26E-23 | 1.42E-21 | 41.3266353 |
| ADAM33 | 1.35894417 | 1.75818395 | 11.4326888 | 5.41E-26 | 1.86E-24 | 48.1424297 |
| VGLL3 | 1.35884731 | 1.80614515 | 17.2571645 | 6.36E-49 | 1.20E-46 | 100.561788 |
| MAP1B | 1.35802576 | 3.60821379 | 11.5171022 | 2.65E-26 | 9.38E-25 | 48.8484309 |
| FABP4 | 1.3578894 | 1.98045441 | 8.6612216 | 1.65E-16 | 2.62E-15 | 26.5408101 |
| FAM180A | 1.35670649 | 1.38294401 | 16.2434839 | 8.73E-45 | 1.22E-42 | 91.0950329 |
| BNC2 | 1.35463134 | 1.54095473 | 16.5315399 | 5.86E-46 | 8.73E-44 | 93.7782795 |
| PDE3A | 1.35367215 | 2.598662 | 11.9107412 | 9.29E-28 | 3.56E-26 | 52.171167 |
| GNAO1 | 1.3529271 | 1.86773637 | 10.0604088 | 4.04E-21 | 9.59E-20 | 37.0305872 |
| COLEC12 | 1.35124681 | 2.04553607 | 13.9279887 | 1.68E-35 | 1.10E-33 | 69.8610249 |
| TSHZ3 | 1.34852942 | 2.55627949 | 16.2646347 | 7.16E-45 | 1.01E-42 | 91.2918311 |
| NGFR | 1.34848484 | 1.90936213 | 8.2682493 | 2.73E-15 | 3.91E-14 | 23.7747369 |
| LOXL1 | 1.34462583 | 4.73619147 | 14.9542767 | 1.41E-39 | 1.29E-37 | 79.1837894 |
| HEYL | 1.34024224 | 4.21105847 | 12.2325207 | 5.79E-29 | 2.45E-27 | 54.922647 |
| TNFAIP6 | 1.33757586 | 3.17940086 | 11.2063927 | 3.61E-25 | 1.17E-23 | 46.2617102 |
| C14orf132 | 1.33716169 | 2.27770883 | 11.8679943 | 1.34E-27 | 5.09E-26 | 51.8079776 |
| GAS7 | 1.33667027 | 3.18176675 | 13.197246 | 1.20E-32 | 6.44E-31 | 63.3406505 |
| FCGR3A | 1.33653049 | 4.93056556 | 9.49489548 | 3.30E-19 | 6.61E-18 | 32.6776579 |
| SVEP1 | 1.33522615 | 2.00003848 | 12.7135572 | 8.67E-31 | 4.06E-29 | 59.0903135 |
| ABI3BP | 1.3337161 | 2.67922178 | 8.87005819 | 3.60E-17 | 6.14E-16 | 28.0448072 |
| DEPP1 | 1.33351834 | 5.79808593 | 11.3673873 | 9.36E-26 | 3.16E-24 | 47.5979146 |
| JAM3 | 1.33184263 | 3.53092243 | 14.4857237 | 1.04E-37 | 8.28E-36 | 74.9063826 |
| COL15A1 | 1.33182674 | 5.88473811 | 12.5522364 | 3.57E-30 | 1.61E-28 | 57.685714 |
| PDZRN3 | 1.33010748 | 3.18710994 | 10.6211345 | 4.49E-23 | 1.22E-21 | 41.4831201 |
| HMCN1 | 1.32756742 | 1.73579796 | 14.8256955 | 4.60E-39 | 4.01E-37 | 78.0067652 |
| GLI3 | 1.3252445 | 1.89621118 | 16.6899603 | 1.32E-46 | 2.19E-44 | 95.2565519 |
| A2M | 1.32498164 | 7.93331962 | 12.2151859 | 6.73E-29 | 2.82E-27 | 54.77365 |
| SLIT3 | 1.32472033 | 2.64150633 | 10.9359735 | 3.40E-24 | 1.00E-22 | 44.037965 |
| OLFML1 | 1.32330477 | 2.99828102 | 17.7826559 | 4.46E-51 | 9.80E-49 | 105.490086 |
| PDLIM7 | 1.32168798 | 5.7106307 | 14.2129242 | 1.26E-36 | 8.95E-35 | 72.4318474 |
| CPED1 | 1.32035731 | 2.77898446 | 9.97561543 | 7.89E-21 | 1.81E-19 | 36.3687797 |
| SPP1 | 1.31746494 | 6.20247191 | 6.11978573 | 2.47E-09 | 1.96E-08 | 10.3234459 |
| PPP1R12B | 1.31134248 | 3.89668264 | 8.47849846 | 6.16E-16 | 9.28E-15 | 25.2440552 |
| RARRES2 | 1.31104101 | 5.66327457 | 14.08157 | 4.17E-36 | 2.85E-34 | 71.244887 |
| PDPN | 1.30934856 | 4.33283697 | 13.9898522 | 9.59E-36 | 6.46E-34 | 70.41793 |
| C8orf88 | 1.30355691 | 1.70639002 | 10.2845914 | 6.79E-22 | 1.70E-20 | 38.795162 |
| AKT3 | 1.30178764 | 2.9830155 | 14.2582375 | 8.35E-37 | 6.08E-35 | 72.8420133 |
| PMP22 | 1.30158191 | 5.71196123 | 15.1285633 | 2.81E-40 | 2.75E-38 | 80.7828068 |
| SYDE1 | 1.30008481 | 3.72748188 | 16.5858569 | 3.52E-46 | 5.49E-44 | 94.2849346 |
| FBXL7 | 1.29662318 | 2.83054085 | 15.6200401 | 2.94E-42 | 3.18E-40 | 85.3123502 |
| CCL19 | 1.29000306 | 3.88337508 | 5.66287489 | 3.07E-08 | 2.14E-07 | 7.87272033 |
| NR2F1 | 1.28998765 | 3.85534334 | 10.5586036 | 7.47E-23 | 1.99E-21 | 40.9802503 |
| EHD2 | 1.28680266 | 6.37404948 | 12.4916805 | 6.07E-30 | 2.68E-28 | 57.1602308 |
| OLR1 | 1.28656929 | 2.69587357 | 10.9540618 | 2.93E-24 | 8.71E-23 | 44.1858812 |
| ZEB1 | 1.28560145 | 3.75752858 | 12.9501661 | 1.07E-31 | 5.33E-30 | 61.1624099 |
| IGFBP7 | 1.28493802 | 9.13664276 | 14.5732359 | 4.68E-38 | 3.78E-36 | 75.7027629 |
| RASSF8 | 1.28302091 | 2.43721401 | 12.7640964 | 5.56E-31 | 2.61E-29 | 59.5317334 |
| ATP8B2 | 1.28177267 | 3.29863339 | 13.0074673 | 6.47E-32 | 3.27E-30 | 61.6662906 |
| TGFBI | 1.28127116 | 5.85084362 | 11.4350741 | 5.30E-26 | 1.83E-24 | 48.1623463 |
| EPHA3 | 1.278755 | 2.24783417 | 10.6781811 | 2.82E-23 | 7.77E-22 | 41.9432224 |
| PHLDA3 | 1.27366374 | 4.77086609 | 11.2932731 | 1.74E-25 | 5.83E-24 | 46.9816764 |
| LGALS1 | 1.27365712 | 8.49706998 | 14.3079001 | 5.31E-37 | 3.95E-35 | 73.2919511 |
| ADH1B | 1.27297026 | 2.16527725 | 6.95294179 | 1.72E-11 | 1.71E-10 | 15.180238 |
| GLIS2 | 1.27283976 | 3.97673967 | 13.5149057 | 6.98E-34 | 4.05E-32 | 66.1614338 |
| GPX8 | 1.269219 | 3.73960596 | 14.9985658 | 9.35E-40 | 8.76E-38 | 79.5897399 |
| MARVELD1 | 1.26910032 | 5.36967212 | 14.4793296 | 1.11E-37 | 8.73E-36 | 74.8482415 |
| TREM2 | 1.26809359 | 4.09929637 | 9.79655492 | 3.21E-20 | 6.99E-19 | 34.981627 |
| TNXB | 1.26808343 | 2.54063828 | 8.8929131 | 3.04E-17 | 5.21E-16 | 28.2107948 |
| ADCY5 | 1.26391146 | 2.24944107 | 8.31729896 | 1.94E-15 | 2.80E-14 | 24.1153034 |
| LAYN | 1.26077441 | 2.543455 | 14.1954311 | 1.48E-36 | 1.04E-34 | 72.273599 |
| MRAS | 1.26062403 | 2.8676545 | 15.7519573 | 8.62E-43 | 1.01E-40 | 86.5327741 |
| SDC2 | 1.25986771 | 4.66034072 | 15.1244417 | 2.92E-40 | 2.84E-38 | 80.744946 |
| HSD11B1 | 1.25737365 | 2.49162852 | 11.88489 | 1.16E-27 | 4.43E-26 | 51.9514617 |
| MXRA7 | 1.25528218 | 4.2074045 | 12.592088 | 2.52E-30 | 1.14E-28 | 58.0320638 |
| PALM | 1.25505595 | 3.0330141 | 10.026584 | 5.28E-21 | 1.24E-19 | 36.7662118 |
| HTRA1 | 1.25285257 | 6.4541811 | 13.5348608 | 5.83E-34 | 3.40E-32 | 66.3393589 |
| GLI2 | 1.25258441 | 2.35114023 | 13.4872429 | 8.95E-34 | 5.12E-32 | 65.9149231 |
| FGL2 | 1.25123391 | 4.51933705 | 8.48779659 | 5.76E-16 | 8.72E-15 | 25.3096024 |
| CMTM3 | 1.25073422 | 4.36954694 | 14.7747121 | 7.36E-39 | 6.28E-37 | 77.540718 |
| RCN3 | 1.24890407 | 5.44041851 | 12.8869452 | 1.88E-31 | 9.16E-30 | 60.6073967 |
| PTGER3 | 1.24879938 | 1.44641691 | 15.0005661 | 9.18E-40 | 8.66E-38 | 79.6080808 |
| MYH10 | 1.24714012 | 3.78925593 | 11.5642235 | 1.78E-26 | 6.36E-25 | 49.2435633 |
| LRRN4CL | 1.24572015 | 2.03922751 | 13.2975638 | 4.89E-33 | 2.67E-31 | 64.2290639 |
| KCNJ8 | 1.24526472 | 3.09083408 | 14.297623 | 5.83E-37 | 4.29E-35 | 73.1988072 |
| PHLDB2 | 1.24216363 | 2.55427496 | 12.1525178 | 1.16E-28 | 4.73E-27 | 54.2357198 |
| NTN1 | 1.2421408 | 3.12586407 | 8.74569634 | 8.94E-17 | 1.46E-15 | 27.1463992 |
| PLSCR4 | 1.2410616 | 3.53081055 | 12.5497052 | 3.65E-30 | 1.64E-28 | 57.6637292 |
| NID2 | 1.24062281 | 3.75382826 | 12.9484645 | 1.09E-31 | 5.40E-30 | 61.147459 |
| PRICKLE2 | 1.24020593 | 2.22135764 | 12.1348892 | 1.35E-28 | 5.49E-27 | 54.0846037 |
| LGI2 | 1.23846721 | 2.09313728 | 11.1971485 | 3.90E-25 | 1.26E-23 | 46.1852598 |
| S1PR3 | 1.23698108 | 3.14151612 | 11.6228094 | 1.08E-26 | 3.92E-25 | 49.7358451 |
| VSIG4 | 1.23486275 | 3.87904307 | 9.2672769 | 1.86E-18 | 3.55E-17 | 30.967606 |
| SNAI2 | 1.23383408 | 3.59760755 | 14.5471644 | 5.95E-38 | 4.74E-36 | 75.4653807 |
| NIBAN1 | 1.2331811 | 4.03816191 | 8.79740634 | 6.13E-17 | 1.02E-15 | 27.518972 |
| P3H3 | 1.22911643 | 3.26360779 | 13.4903747 | 8.70E-34 | 5.00E-32 | 65.9428231 |
| GALNT15 | 1.22905057 | 1.40937386 | 15.4429461 | 1.53E-41 | 1.57E-39 | 83.6769455 |
| CSDC2 | 1.22149465 | 1.43105407 | 16.5788141 | 3.76E-46 | 5.73E-44 | 94.2192296 |
| DNAJB5 | 1.22114424 | 2.5817115 | 10.7652666 | 1.39E-23 | 3.91E-22 | 42.6480291 |
| RERG | 1.22058091 | 2.33284285 | 9.99506246 | 6.77E-21 | 1.57E-19 | 36.5202859 |
| NCS1 | 1.22023777 | 3.79763284 | 9.55444694 | 2.09E-19 | 4.28E-18 | 33.1291414 |
| WWTR1 | 1.22019558 | 4.68897162 | 11.7619599 | 3.31E-27 | 1.23E-25 | 50.9095211 |
| IL1R1 | 1.21997475 | 4.75613663 | 13.8057542 | 5.08E-35 | 3.24E-33 | 68.7627759 |
| ECM2 | 1.21618506 | 2.6316586 | 16.1659111 | 1.80E-44 | 2.37E-42 | 90.3735714 |
| GLI1 | 1.2159077 | 1.85017572 | 13.5877402 | 3.63E-34 | 2.16E-32 | 66.8112481 |
| KIRREL1 | 1.21334025 | 3.99998264 | 13.7230253 | 1.07E-34 | 6.62E-33 | 68.0211194 |
| MAFB | 1.21133771 | 4.84423735 | 11.9583217 | 6.17E-28 | 2.41E-26 | 52.5760798 |
| NPTXR | 1.21114961 | 2.31815534 | 8.91902291 | 2.51E-17 | 4.34E-16 | 28.4007535 |
| FMOD | 1.20875881 | 6.04528084 | 9.50504594 | 3.05E-19 | 6.14E-18 | 32.7544943 |
| MSC | 1.20803407 | 3.66844818 | 13.8821792 | 2.54E-35 | 1.66E-33 | 69.4491023 |
| HIC1 | 1.20773068 | 3.02320883 | 13.8312915 | 4.03E-35 | 2.60E-33 | 68.9919858 |
| CNTNAP1 | 1.20752383 | 2.9325702 | 12.1340063 | 1.36E-28 | 5.52E-27 | 54.0770372 |
| ZNF423 | 1.20741302 | 1.9035973 | 13.6300166 | 2.48E-34 | 1.49E-32 | 67.1889318 |
| MPDZ | 1.20604608 | 2.03843653 | 13.5697424 | 4.26E-34 | 2.53E-32 | 66.6505724 |
| SIX2 | 1.20348828 | 2.52918275 | 6.23007253 | 1.32E-09 | 1.08E-08 | 10.9381318 |
| DKK3 | 1.2034761 | 5.19420705 | 13.2911933 | 5.18E-33 | 2.82E-31 | 64.17258 |
| FOXS1 | 1.20231169 | 3.21417513 | 11.5514486 | 1.98E-26 | 7.05E-25 | 49.1363687 |
| GPR68 | 1.20224605 | 3.01312656 | 15.4471988 | 1.47E-41 | 1.53E-39 | 83.7161763 |
| FILIP1L | 1.20133463 | 5.16807145 | 11.9208082 | 8.52E-28 | 3.28E-26 | 52.2567803 |
| LDOC1 | 1.1989748 | 3.97345807 | 9.37966613 | 7.95E-19 | 1.55E-17 | 31.8088412 |
| FZD8 | 1.198841 | 3.68795283 | 11.1908018 | 4.11E-25 | 1.33E-23 | 46.1327901 |
| ITGA5 | 1.19698299 | 5.41566681 | 11.4463328 | 4.82E-26 | 1.67E-24 | 48.2563814 |
| ADAMTS1 | 1.19262335 | 3.94730027 | 10.5443336 | 8.38E-23 | 2.22E-21 | 40.8657089 |
| LRRC17 | 1.18919983 | 1.71834502 | 14.8549748 | 3.51E-39 | 3.14E-37 | 78.2745808 |
| FOXF1 | 1.1879896 | 4.24317557 | 9.861871 | 1.93E-20 | 4.29E-19 | 35.4859661 |
| ARSI | 1.18566475 | 1.63986476 | 13.9880663 | 9.74E-36 | 6.49E-34 | 70.4018428 |
| EML1 | 1.18560449 | 2.61389559 | 11.4965063 | 3.16E-26 | 1.11E-24 | 48.6759563 |
| FAM20C | 1.18505218 | 4.82003221 | 11.9208937 | 8.51E-28 | 3.28E-26 | 52.2575077 |
| CLIC4 | 1.18379736 | 6.49171032 | 13.1607458 | 1.66E-32 | 8.81E-31 | 63.0179718 |
| PCDH7 | 1.18271272 | 3.18164279 | 8.30594319 | 2.10E-15 | 3.03E-14 | 24.036336 |
| SGCA | 1.18202115 | 1.97425741 | 8.70361745 | 1.21E-16 | 1.95E-15 | 26.8442649 |
| SH3PXD2B | 1.18050179 | 4.43370203 | 14.4607756 | 1.31E-37 | 1.03E-35 | 74.6795714 |
| TENM3 | 1.18005251 | 1.26797608 | 13.3111753 | 4.33E-33 | 2.38E-31 | 64.3497806 |
| KANK2 | 1.17832809 | 4.7883216 | 11.4162705 | 6.21E-26 | 2.13E-24 | 48.00539 |
| HEG1 | 1.17788545 | 4.44917341 | 12.8631308 | 2.32E-31 | 1.12E-29 | 60.3985853 |
| CFH | 1.17748475 | 4.16477495 | 10.1596676 | 1.84E-21 | 4.47E-20 | 37.8092383 |
| SPON2 | 1.17685652 | 4.87699662 | 12.0094455 | 3.98E-28 | 1.59E-26 | 53.0119079 |
| LAMA4 | 1.17679373 | 4.47780614 | 13.6791236 | 1.59E-34 | 9.70E-33 | 67.6280955 |
| LZTS1 | 1.17263401 | 2.66410363 | 13.3449125 | 3.20E-33 | 1.78E-31 | 64.6491632 |
| SYT11 | 1.17235601 | 3.38594233 | 11.4161009 | 6.22E-26 | 2.13E-24 | 48.0039749 |
| MATN3 | 1.17227982 | 2.19961162 | 9.36305959 | 9.02E-19 | 1.76E-17 | 31.6841549 |
| AMOTL1 | 1.17017643 | 3.66016072 | 10.4466281 | 1.85E-22 | 4.78E-21 | 40.0836332 |
| FMO2 | 1.16754735 | 2.07193864 | 8.60945838 | 2.40E-16 | 3.77E-15 | 26.1716157 |
| PSD | 1.16388288 | 1.90888091 | 8.8477524 | 4.24E-17 | 7.19E-16 | 27.8830698 |
| AMIGO2 | 1.16386205 | 3.42245431 | 9.8943722 | 1.49E-20 | 3.37E-19 | 35.7376355 |
| SCG2 | 1.16363596 | 1.73141701 | 8.97016341 | 1.72E-17 | 3.03E-16 | 28.7738399 |
| CXCL12 | 1.16271861 | 3.77282861 | 9.25097191 | 2.11E-18 | 3.99E-17 | 30.8460752 |
| RAB23 | 1.1624577 | 3.04303083 | 11.0040251 | 1.94E-24 | 5.84E-23 | 44.5950814 |
| SLC22A17 | 1.16178255 | 2.54928676 | 10.7280342 | 1.88E-23 | 5.26E-22 | 42.3463401 |
| ZNF521 | 1.16042828 | 2.22954739 | 15.1960986 | 1.51E-40 | 1.50E-38 | 81.4034904 |
| CCL11 | 1.15938435 | 4.30818711 | 7.3105145 | 1.77E-12 | 1.94E-11 | 17.4095669 |
| ID4 | 1.15841383 | 3.56386374 | 10.5190327 | 1.03E-22 | 2.69E-21 | 40.6628235 |
| GYPC | 1.15816236 | 3.90914121 | 11.0346927 | 1.50E-24 | 4.59E-23 | 44.8467009 |
| CRISPLD1 | 1.15803296 | 2.25004286 | 10.4862685 | 1.34E-22 | 3.50E-21 | 40.4004699 |
| MYOCD | 1.15801664 | 1.85163795 | 8.05312804 | 1.23E-14 | 1.65E-13 | 22.2973191 |
| MMP14 | 1.15457638 | 7.25880559 | 13.0748552 | 3.56E-32 | 1.83E-30 | 62.2598696 |
| EPDR1 | 1.15439591 | 3.33484164 | 9.87447402 | 1.75E-20 | 3.90E-19 | 35.5835003 |
| POPDC2 | 1.15399512 | 2.15042431 | 9.03635803 | 1.05E-17 | 1.90E-16 | 29.2587425 |
| GNB4 | 1.15323375 | 3.56900849 | 12.7845234 | 4.64E-31 | 2.21E-29 | 59.71033 |
| TCEAL7 | 1.152766 | 1.97068441 | 13.2842604 | 5.51E-33 | 2.99E-31 | 64.1111189 |
| CAV1 | 1.15257724 | 5.33041021 | 9.72136036 | 5.76E-20 | 1.23E-18 | 34.4033962 |
| NXN | 1.15187175 | 3.8115208 | 11.0181077 | 1.73E-24 | 5.26E-23 | 44.7105826 |
| ARMCX2 | 1.14578353 | 3.08814569 | 11.2798682 | 1.95E-25 | 6.44E-24 | 46.8704208 |
| AOX1 | 1.14561802 | 1.48901727 | 10.7686554 | 1.35E-23 | 3.81E-22 | 42.6755143 |
| SVIL | 1.14540686 | 4.83709757 | 9.10453514 | 6.33E-18 | 1.17E-16 | 29.7604949 |
| VEGFC | 1.14449276 | 3.22784215 | 16.5992695 | 3.11E-46 | 4.90E-44 | 94.4100762 |
| PRSS23 | 1.14406246 | 4.23822624 | 13.0391912 | 4.88E-32 | 2.48E-30 | 61.9455928 |
| NLGN2 | 1.14223549 | 3.52336994 | 12.2322484 | 5.81E-29 | 2.45E-27 | 54.9203063 |
| MEIS3 | 1.14022029 | 2.16918297 | 16.2256959 | 1.03E-44 | 1.41E-42 | 90.9295516 |
| EFS | 1.13991857 | 2.30867919 | 12.1994439 | 7.71E-29 | 3.20E-27 | 54.638417 |
| KCTD12 | 1.13693501 | 5.79046976 | 9.77918786 | 3.67E-20 | 7.98E-19 | 34.8478501 |
| SHISAL1 | 1.13640799 | 1.45376736 | 9.29289556 | 1.54E-18 | 2.94E-17 | 31.1588206 |
| CD109 | 1.13562324 | 2.54899939 | 10.1178041 | 2.56E-21 | 6.16E-20 | 37.4803188 |
| FRZB | 1.13513742 | 3.79749437 | 8.02209049 | 1.52E-14 | 2.03E-13 | 22.0863697 |
| TMOD1 | 1.13505768 | 1.96809478 | 8.95848903 | 1.87E-17 | 3.29E-16 | 28.688553 |
| TUBB6 | 1.13337152 | 4.74279057 | 11.4769055 | 3.72E-26 | 1.30E-24 | 48.5119448 |
| PKD2 | 1.1328716 | 3.87428787 | 14.3880685 | 2.55E-37 | 1.92E-35 | 74.0191431 |
| MSR1 | 1.13206953 | 2.99199817 | 10.5216902 | 1.01E-22 | 2.64E-21 | 40.6841213 |
| JAM2 | 1.13111076 | 2.56708181 | 10.5874644 | 5.91E-23 | 1.58E-21 | 41.2121558 |
| GLIPR1 | 1.12699347 | 3.39398121 | 11.8074808 | 2.25E-27 | 8.41E-26 | 51.2948028 |
| TIMP1 | 1.11977975 | 9.7271049 | 11.9976198 | 4.40E-28 | 1.74E-26 | 52.9110242 |
| FPR3 | 1.11884908 | 4.24869367 | 8.80572556 | 5.77E-17 | 9.63E-16 | 27.579044 |
| NREP | 1.1165197 | 3.63563584 | 14.0670517 | 4.76E-36 | 3.24E-34 | 71.113884 |
| C4A | 1.1146519 | 2.51854038 | 9.51766958 | 2.77E-19 | 5.58E-18 | 32.85012 |
| C1QTNF3 | 1.11459086 | 2.06283252 | 11.2055173 | 3.63E-25 | 1.18E-23 | 46.2544695 |
| EGR2 | 1.11335129 | 2.86690804 | 9.52135815 | 2.69E-19 | 5.43E-18 | 32.8780756 |
| PLPPR4 | 1.11307903 | 1.56840187 | 13.0622098 | 3.98E-32 | 2.03E-30 | 62.1484026 |
| PDLIM4 | 1.11256444 | 3.76348592 | 9.88046245 | 1.67E-20 | 3.73E-19 | 35.6298693 |
| PDGFC | 1.1115367 | 3.0199088 | 14.852538 | 3.59E-39 | 3.17E-37 | 78.2522874 |
| ITGA7 | 1.11046545 | 3.27254251 | 8.73420619 | 9.73E-17 | 1.58E-15 | 27.0638043 |
| LSAMP | 1.1096677 | 2.08999197 | 11.3519489 | 1.07E-25 | 3.59E-24 | 47.4693937 |
| C5AR1 | 1.10949076 | 4.13519581 | 10.1103467 | 2.72E-21 | 6.51E-20 | 37.421805 |
| ANXA6 | 1.10875476 | 5.37835861 | 11.0071958 | 1.89E-24 | 5.70E-23 | 44.6210801 |
| CACNA1C | 1.10864746 | 1.88656159 | 10.0580099 | 4.12E-21 | 9.75E-20 | 37.0118207 |
| RECK | 1.10795864 | 2.28235463 | 14.7696949 | 7.70E-39 | 6.54E-37 | 77.4948753 |
| VIM | 1.10759603 | 7.87471043 | 13.7822608 | 6.28E-35 | 3.95E-33 | 68.5520234 |
| P4HA3 | 1.10720726 | 1.60347406 | 17.5609208 | 3.62E-50 | 7.35E-48 | 103.409332 |
| DAAM2 | 1.10382599 | 2.7998373 | 9.46491518 | 4.15E-19 | 8.26E-18 | 32.4509999 |
| FZD1 | 1.10291734 | 3.79398127 | 14.1212761 | 2.91E-36 | 2.01E-34 | 71.6033627 |
| PTPRS | 1.10286243 | 3.29139873 | 8.46497154 | 6.78E-16 | 1.02E-14 | 25.1487823 |
| ADAMTS8 | 1.09913647 | 1.6518995 | 8.52267549 | 4.49E-16 | 6.87E-15 | 25.5559043 |
| CFD | 1.09817991 | 5.42936306 | 7.65566814 | 1.83E-13 | 2.20E-12 | 19.6392554 |
| CACNA2D1 | 1.09620374 | 2.36420983 | 9.7535764 | 4.48E-20 | 9.64E-19 | 34.6508165 |
| RCAN2 | 1.09556585 | 3.67315902 | 8.99926701 | 1.39E-17 | 2.47E-16 | 28.98676 |
| SELENOM | 1.0946715 | 5.49534386 | 10.2391739 | 9.76E-22 | 2.41E-20 | 38.4359601 |
| PRKG1 | 1.09459862 | 2.31720862 | 12.9904253 | 7.52E-32 | 3.76E-30 | 61.5163487 |
| IGFBP3 | 1.09454483 | 6.7595257 | 9.6699924 | 8.57E-20 | 1.82E-18 | 34.00987 |
| ANTXR2 | 1.09310348 | 4.13028861 | 10.2252657 | 1.09E-21 | 2.69E-20 | 38.3261337 |
| SGCE | 1.09253716 | 3.74005218 | 9.66329504 | 9.03E-20 | 1.92E-18 | 33.9586513 |
| CSRP1 | 1.09248305 | 6.08686762 | 9.89269556 | 1.51E-20 | 3.40E-19 | 35.7246412 |
| SORCS2 | 1.0916125 | 1.52380619 | 12.6667596 | 1.31E-30 | 6.05E-29 | 58.6821566 |
| ZNF469 | 1.09110197 | 2.13648837 | 11.5692487 | 1.71E-26 | 6.11E-25 | 49.2857454 |
| RAI2 | 1.09081562 | 3.86818624 | 8.62719006 | 2.11E-16 | 3.33E-15 | 26.297922 |
| PRKD1 | 1.09047737 | 1.60852504 | 16.1120162 | 2.99E-44 | 3.85E-42 | 89.8726268 |
| SH3RF3 | 1.09013923 | 2.77635194 | 12.9652392 | 9.40E-32 | 4.69E-30 | 61.2948796 |
| RNF144A | 1.08995635 | 3.11473121 | 13.4300323 | 1.49E-33 | 8.45E-32 | 65.4056185 |
| CES1 | 1.08938608 | 2.66570268 | 5.75089569 | 1.91E-08 | 1.37E-07 | 8.33263418 |
| CAVIN2 | 1.08807663 | 3.60813927 | 7.78167005 | 7.84E-14 | 9.84E-13 | 20.4716322 |
| SPEG | 1.08798242 | 1.62361253 | 8.1858741 | 4.87E-15 | 6.80E-14 | 23.2058554 |
| DUSP1 | 1.0867496 | 7.66603577 | 9.02500881 | 1.14E-17 | 2.06E-16 | 29.175446 |
| KIAA1755 | 1.08674381 | 1.90443184 | 14.3970962 | 2.35E-37 | 1.78E-35 | 74.1010987 |
| COX7A1 | 1.08630511 | 3.77795313 | 12.9313704 | 1.27E-31 | 6.25E-30 | 60.9973018 |
| NAP1L3 | 1.08417689 | 1.23434276 | 15.5663386 | 4.85E-42 | 5.16E-40 | 84.8160661 |
| DCHS1 | 1.08244448 | 3.47801849 | 11.1157761 | 7.67E-25 | 2.39E-23 | 45.5136103 |
| RBMS3 | 1.08000712 | 1.98302452 | 12.4710833 | 7.26E-30 | 3.18E-28 | 56.9817194 |
| CDK14 | 1.07845097 | 2.72332624 | 11.1888033 | 4.18E-25 | 1.35E-23 | 46.116271 |
| UCHL1 | 1.07834592 | 2.81299233 | 6.89282933 | 2.50E-11 | 2.44E-10 | 14.8137579 |
| DIXDC1 | 1.07608372 | 2.9048286 | 10.3021998 | 5.90E-22 | 1.49E-20 | 38.934656 |
| SMAD9 | 1.07446733 | 2.37445605 | 8.32708523 | 1.81E-15 | 2.63E-14 | 24.1834147 |
| CSF1R | 1.07357852 | 4.44823881 | 8.60771262 | 2.43E-16 | 3.82E-15 | 26.1591895 |
| RASL12 | 1.07334305 | 3.33403665 | 11.2890288 | 1.81E-25 | 6.03E-24 | 46.9464437 |
| C3AR1 | 1.07298216 | 4.00284181 | 9.04841014 | 9.61E-18 | 1.74E-16 | 29.3472693 |
| CHST3 | 1.07203098 | 2.97996197 | 9.81159072 | 2.85E-20 | 6.25E-19 | 35.0975563 |
| MFGE8 | 1.07039269 | 5.31706927 | 13.1942708 | 1.23E-32 | 6.59E-31 | 63.3143375 |
| NHSL2 | 1.07013902 | 1.50058667 | 11.6262938 | 1.05E-26 | 3.82E-25 | 49.7651587 |
| ALDH1A3 | 1.06900011 | 3.0878921 | 9.77236359 | 3.87E-20 | 8.40E-19 | 34.7953206 |
| GASK1B | 1.06777166 | 4.32463258 | 11.1526204 | 5.65E-25 | 1.77E-23 | 45.8174326 |
| CAP2 | 1.06702912 | 2.90728689 | 8.74515192 | 8.98E-17 | 1.46E-15 | 27.1424841 |
| GJA1 | 1.06661651 | 5.22523788 | 10.050773 | 4.36E-21 | 1.03E-19 | 36.9552227 |
| WNT9A | 1.06374342 | 1.73163774 | 9.10860856 | 6.14E-18 | 1.13E-16 | 29.7905476 |
| PXDN | 1.06343916 | 4.35416348 | 11.5600917 | 1.84E-26 | 6.57E-25 | 49.2088879 |
| DLC1 | 1.06117287 | 3.28270175 | 12.3298168 | 2.49E-29 | 1.08E-27 | 55.7605142 |
| RRAD | 1.05973613 | 3.10807096 | 7.45266334 | 7.01E-13 | 7.94E-12 | 18.3187776 |
| CDC42EP3 | 1.05750415 | 3.3274773 | 10.6457265 | 3.68E-23 | 1.00E-21 | 41.681308 |
| FCGR2A | 1.05709152 | 4.31755253 | 9.69158338 | 7.25E-20 | 1.55E-18 | 34.1751294 |
| ARL4C | 1.05696029 | 4.82741155 | 10.755386 | 1.50E-23 | 4.23E-22 | 42.5679166 |
| SELP | 1.05679559 | 2.63692242 | 7.66525139 | 1.71E-13 | 2.07E-12 | 19.7022222 |
| PDGFRA | 1.05553565 | 3.85980431 | 9.35159473 | 9.84E-19 | 1.91E-17 | 31.5981514 |
| TPM1 | 1.0552808 | 5.63890464 | 10.3980699 | 2.73E-22 | 6.95E-21 | 39.6963845 |
| CAVIN3 | 1.05293391 | 5.79143432 | 11.1370743 | 6.43E-25 | 2.01E-23 | 45.6891787 |
| FMO1 | 1.05043605 | 1.51837504 | 12.5370886 | 4.08E-30 | 1.82E-28 | 57.5541748 |
| ITGA9 | 1.05039988 | 2.91640988 | 9.08891489 | 7.11E-18 | 1.31E-16 | 29.6453296 |
| PDE1A | 1.04948995 | 1.83122919 | 13.0858521 | 3.22E-32 | 1.67E-30 | 62.3568367 |
| GREM2 | 1.04929145 | 1.91499386 | 6.77183034 | 5.27E-11 | 4.97E-10 | 14.0834721 |
| TNS2 | 1.04889533 | 4.49494877 | 11.2829238 | 1.90E-25 | 6.29E-24 | 46.8957757 |
| KLF9 | 1.04886628 | 4.73218001 | 10.1414331 | 2.13E-21 | 5.14E-20 | 37.6658792 |
| RUNX1T1 | 1.04630329 | 1.31876246 | 13.0859738 | 3.22E-32 | 1.67E-30 | 62.3579101 |
| ZFPM2 | 1.04554328 | 1.37968572 | 14.2953558 | 5.95E-37 | 4.36E-35 | 73.1782619 |
| TMEM200B | 1.04480027 | 2.0384444 | 12.0090801 | 3.99E-28 | 1.59E-26 | 53.00879 |
| FILIP1 | 1.04417977 | 1.93329276 | 9.25437866 | 2.05E-18 | 3.90E-17 | 30.8714569 |
| CD163 | 1.04305809 | 3.46367486 | 7.88496077 | 3.89E-14 | 5.01E-13 | 21.1611449 |
| PCDHGB7 | 1.04202622 | 1.71208062 | 13.9876115 | 9.79E-36 | 6.49E-34 | 70.3977465 |
| MMP19 | 1.03978061 | 2.93906484 | 12.3090916 | 2.98E-29 | 1.27E-27 | 55.5818154 |
| ZNF853 | 1.03890281 | 2.40424075 | 8.77207421 | 7.38E-17 | 1.22E-15 | 27.3362764 |
| NUPR1 | 1.03495051 | 3.96706942 | 9.07838892 | 7.69E-18 | 1.41E-16 | 29.5677929 |
| VASN | 1.03352656 | 4.45236034 | 11.0639192 | 1.18E-24 | 3.65E-23 | 45.0868148 |
| PLPP7 | 1.03273052 | 1.56081976 | 13.7977574 | 5.46E-35 | 3.47E-33 | 68.6910268 |
| CNRIP1 | 1.03196896 | 2.6781839 | 13.9630001 | 1.22E-35 | 8.08E-34 | 70.1761158 |
| DCLK2 | 1.03006706 | 1.66454511 | 12.1815902 | 9.00E-29 | 3.71E-27 | 54.4851304 |
| PBX3 | 1.02887348 | 3.86371017 | 11.8184629 | 2.05E-27 | 7.68E-26 | 51.3878498 |
| ATP2B4 | 1.02815846 | 5.29794001 | 9.3285046 | 1.17E-18 | 2.26E-17 | 31.4251346 |
| C1QTNF1 | 1.02735858 | 4.3773839 | 8.89417319 | 3.01E-17 | 5.16E-16 | 28.2199543 |
| PDK4 | 1.02616066 | 4.5071629 | 5.65804278 | 3.15E-08 | 2.20E-07 | 7.84764341 |
| BMERB1 | 1.02602048 | 3.21439776 | 10.0867056 | 3.28E-21 | 7.83E-20 | 37.2364646 |
| C4B | 1.02573601 | 2.50553721 | 8.51455441 | 4.76E-16 | 7.25E-15 | 25.4984966 |
| CSPG4 | 1.02559927 | 3.88580401 | 8.16835829 | 5.51E-15 | 7.64E-14 | 23.0853917 |
| SMIM3 | 1.02280169 | 4.35989109 | 11.9494426 | 6.66E-28 | 2.59E-26 | 52.5004656 |
| SMTN | 1.02247564 | 4.92388376 | 8.56134216 | 3.40E-16 | 5.26E-15 | 25.8297322 |
| ADAMTS10 | 1.02079224 | 1.99828162 | 13.6014531 | 3.21E-34 | 1.92E-32 | 66.9337141 |
| A4GALT | 1.02050128 | 3.68885936 | 10.073354 | 3.65E-21 | 8.67E-20 | 37.1318981 |
| CYP7B1 | 1.02006753 | 1.9857962 | 10.5822769 | 6.16E-23 | 1.65E-21 | 41.1704483 |
| ARHGEF17 | 1.0200492 | 3.92132151 | 11.2883051 | 1.82E-25 | 6.05E-24 | 46.9404365 |
| ZEB2 | 1.01971194 | 2.48291607 | 12.6501606 | 1.51E-30 | 6.95E-29 | 58.5375198 |
| COL4A2 | 1.0189341 | 7.35161141 | 10.26845 | 7.73E-22 | 1.93E-20 | 38.6674031 |
| DOK5 | 1.01892558 | 1.52934471 | 15.4419064 | 1.54E-41 | 1.58E-39 | 83.6673549 |
| RTL8B | 1.01879585 | 3.60060388 | 10.7124795 | 2.13E-23 | 5.95E-22 | 42.22046 |
| NOTCH3 | 1.01839628 | 5.86429183 | 10.1106079 | 2.72E-21 | 6.51E-20 | 37.4238544 |
| DIO2 | 1.01813391 | 3.74090736 | 9.08344284 | 7.41E-18 | 1.36E-16 | 29.6050143 |
| SCARA3 | 1.01783162 | 3.55070173 | 7.14347552 | 5.17E-12 | 5.44E-11 | 16.3577263 |
| PLXNC1 | 1.01756747 | 2.9199212 | 10.9521821 | 2.98E-24 | 8.83E-23 | 44.170505 |
| RFTN1 | 1.017452 | 4.20261056 | 11.1760762 | 4.64E-25 | 1.48E-23 | 46.0111033 |
| JCAD | 1.01577311 | 3.35646143 | 11.0498985 | 1.33E-24 | 4.09E-23 | 44.971588 |
| DKK2 | 1.01546383 | 1.45455168 | 11.0038143 | 1.94E-24 | 5.84E-23 | 44.5933527 |
| SERPINE2 | 1.01502392 | 3.307968 | 8.26818318 | 2.74E-15 | 3.91E-14 | 23.7742788 |
| SEMA3C | 1.01482538 | 4.61785653 | 7.79621348 | 7.11E-14 | 8.95E-13 | 20.568328 |
| COL18A1 | 1.01349158 | 5.89546497 | 10.0377514 | 4.83E-21 | 1.14E-19 | 36.8534413 |
| APOE | 1.01331374 | 7.74040673 | 6.15903161 | 1.98E-09 | 1.59E-08 | 10.5411692 |
| TENM4 | 1.01305502 | 1.24956836 | 14.9203351 | 1.92E-39 | 1.75E-37 | 78.8728647 |
| PCDHGC3 | 1.01213007 | 3.06866967 | 9.29814496 | 1.48E-18 | 2.83E-17 | 31.1980411 |
| ADAM19 | 1.01106298 | 3.71629463 | 11.2582823 | 2.34E-25 | 7.68E-24 | 46.6913958 |
| NOX4 | 1.01081217 | 1.25835586 | 20.2825029 | 2.44E-61 | 1.63E-58 | 128.970844 |
| CH25H | 1.00981803 | 2.55666134 | 8.62933803 | 2.08E-16 | 3.28E-15 | 26.3132339 |
| ENOX1 | 1.00937164 | 1.67359575 | 13.341698 | 3.30E-33 | 1.83E-31 | 64.6206272 |
| ITGA1 | 1.00912124 | 3.9734094 | 10.6114939 | 4.86E-23 | 1.31E-21 | 41.4054906 |
| CTSO | 1.00880116 | 4.83388155 | 12.0004812 | 4.30E-28 | 1.70E-26 | 52.9354304 |
| CCL2 | 1.00861479 | 5.102223 | 8.25275991 | 3.05E-15 | 4.33E-14 | 23.6674725 |
| MS4A4A | 1.00855096 | 3.35233105 | 8.60234693 | 2.53E-16 | 3.96E-15 | 26.1210071 |
| BVES | 1.00852058 | 1.82994834 | 8.4692318 | 6.58E-16 | 9.89E-15 | 25.1787774 |
| NFATC4 | 1.00762172 | 2.2778418 | 12.3056453 | 3.07E-29 | 1.31E-27 | 55.5521127 |
| ASB2 | 1.00754165 | 2.27255126 | 6.98995113 | 1.36E-11 | 1.38E-10 | 15.4070708 |
| CCDC69 | 1.00569758 | 4.11593944 | 7.94147889 | 2.64E-14 | 3.46E-13 | 21.5411292 |
| RHOJ | 1.00339878 | 3.36518276 | 11.287271 | 1.83E-25 | 6.08E-24 | 46.9318535 |
| PLEKHO1 | 1.00262635 | 3.85776775 | 10.4779845 | 1.43E-22 | 3.73E-21 | 40.3342056 |
| TNFSF4 | 1.00240151 | 1.871275 | 13.0860856 | 3.22E-32 | 1.67E-30 | 62.3588958 |
| RAB3IL1 | 1.00214958 | 3.3255591 | 10.8211759 | 8.76E-24 | 2.50E-22 | 43.1020476 |
| DZIP1 | 1.00014534 | 1.75191041 | 12.7758732 | 5.01E-31 | 2.37E-29 | 59.6346873 |
| REG3A | -1.0160424 | 3.85090018 | -2.7522812 | 0.00622123 | 0.01663462 | -3.7256693 |
| DUOXA2 | -1.0323446 | 3.01523756 | -4.2225854 | 3.07E-05 | 0.00014027 | 1.22317879 |
| PRSS2 | -1.0445314 | 3.6036184 | -3.3354269 | 0.00094139 | 0.00314124 | -1.9968586 |
| AKR1B10 | -1.075826 | 5.36330511 | -3.571253 | 0.0004041 | 0.00147749 | -1.2092733 |
| LCN2 | -1.075899 | 9.32719 | -3.9287421 | 0.00010258 | 0.00042674 | 0.07961972 |
| CCL20 | -1.1018099 | 5.51070336 | -4.8067286 | 2.27E-06 | 1.25E-05 | 3.71566424 |
| BPIFB1 | -1.1647471 | 4.36321398 | -3.4157077 | 0.00070962 | 0.00244715 | -1.7343954 |
