# Supplementary material for: Molecular Typing of Gastric Cancer Based on Invasion-Related Genes and Prognosis-Related Features
Source: Front Oncol. 2022 Jun 3;12:848163. doi: 10.3389/fonc.2022.848163 (PMC9203697; doi:10.3389/fonc.2022.848163)
Supplement: Supplementary file 3 [file Table_3.docx]

Table S3. Univariate Cox regression analysis on the TCGA training set data for the DEGs between C1 and C2 subtypes

| Name | p.value | HR | Low 95%CI | High 95%CI |
| --- | --- | --- | --- | --- |
| COL10A1 | 0.0451505 | 1.1230318 | 1.00251033 | 1.25804232 |
| COL11A1 | 0.03300306 | 1.14789224 | 1.01119462 | 1.30306923 |
| CTHRC1 | 0.01609741 | 1.22467803 | 1.03833806 | 1.44445855 |
| POSTN | 0.02271114 | 1.20614162 | 1.02653117 | 1.41717821 |
| INHBA | 0.02187333 | 1.23842563 | 1.0315095 | 1.48684821 |
| VCAN | 0.02140553 | 1.24285376 | 1.03272716 | 1.49573435 |
| FAP | 0.02627807 | 1.25505358 | 1.02715996 | 1.53350945 |
| MMP11 | 0.02325086 | 1.15141776 | 1.01940456 | 1.3005267 |
| SPARC | 0.01635854 | 1.29114146 | 1.04803959 | 1.59063293 |
| COL5A2 | 0.02834046 | 1.24280327 | 1.02332568 | 1.50935328 |
| LOX | 0.00727073 | 1.28387236 | 1.069745 | 1.5408609 |
| CST2 | 0.02524326 | 1.17133378 | 1.01981955 | 1.34535843 |
| ADAMTS12 | 0.01616428 | 1.25765214 | 1.04334883 | 1.51597323 |
| ADAM12 | 0.04043222 | 1.1977454 | 1.00789755 | 1.42335304 |
| MFAP2 | 0.03119411 | 1.2044349 | 1.01694426 | 1.42649256 |
| EDNRA | 0.02337048 | 1.28336263 | 1.03439853 | 1.59224864 |
| GPNMB | 0.03319869 | 1.19746221 | 1.01446364 | 1.4134718 |
| WNT2 | 0.01286629 | 1.23681353 | 1.0461017 | 1.46229349 |
| SERPINE1 | 0.00125454 | 1.27340427 | 1.09950535 | 1.47480724 |
| TNFAIP6 | 0.04643945 | 1.22159704 | 1.00316241 | 1.48759495 |
| OLR1 | 0.02538241 | 1.21888123 | 1.02469885 | 1.44986154 |
| GPX8 | 0.04329368 | 1.2680608 | 1.00718273 | 1.59651088 |
| NID2 | 0.0097853 | 1.34355219 | 1.07386098 | 1.6809741 |
| MATN3 | 0.00309261 | 1.3338738 | 1.10210924 | 1.61437655 |
| AMIGO2 | 0.00410061 | 1.31836375 | 1.09161757 | 1.59220869 |
| CD109 | 0.02017459 | 1.22240598 | 1.03188817 | 1.44809915 |
| NREP | 0.02730176 | 1.31853905 | 1.03145639 | 1.6855247 |
| RNF144A | 0.04786654 | 1.28450368 | 1.00235487 | 1.64607342 |
| GASK1B | 0.03210964 | 1.26103847 | 1.02001174 | 1.55901933 |
| PXDN | 0.03913168 | 1.27792334 | 1.01230066 | 1.61324411 |
| TENM4 | 0.03517971 | 1.34904287 | 1.02101626 | 1.78245609 |
| NOX4 | 0.00192002 | 1.72762921 | 1.22302737 | 2.44042181 |
