# Supplementary figures and images for: Molecular Typing of Gastric Cancer Based on Invasion-Related Genes and Prognosis-Related Features
Source: Front Oncol. 2022 Jun 3;12:848163. doi: 10.3389/fonc.2022.848163 (PMC9203697; doi:10.3389/fonc.2022.848163)

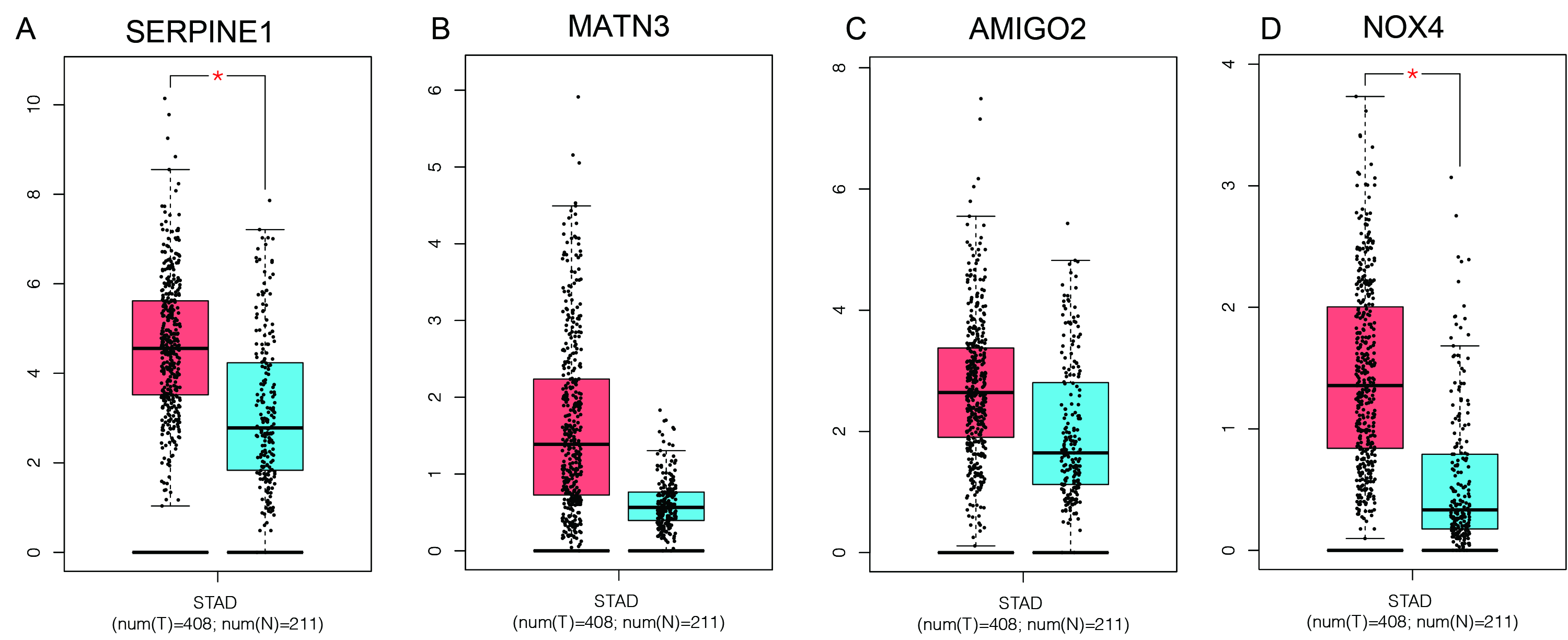

Supplement: Supplementary Figure 1 — Comparison of the expression of four genes between tumour and healthy samples included in TCGA dataset. [file Image_1.tif]

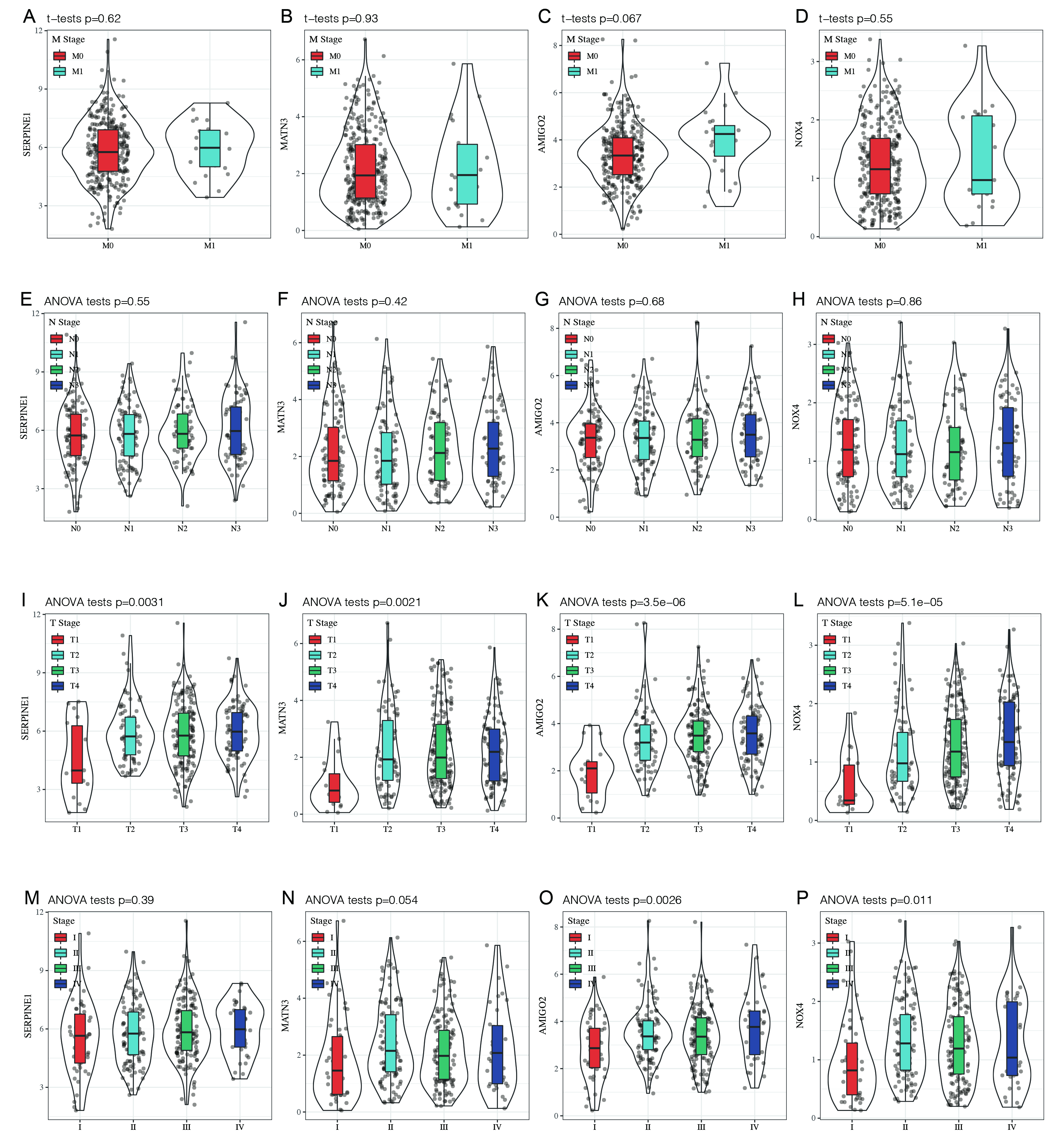

Supplement: Supplementary Figure 2 — Comparison of the expression of four genes based on the TNM classification. [file Image_2.tif]
